# Alzheimer's Protection by PLCγ2 Compacts Plaques, Redistributes Microglia, and Protects Synapses in *App* ^*NL*^ ^*‐G‐F*^ Mice

**DOI:** 10.1002/glia.70192

**Published:** 2026-06-25

**Authors:** Ryan J. Bevan, Emily Maguire, Eilish Mackinnon, Elisa Salis, Thomas Phillips, Elena Simonazzi, Marieta Vassileva, Nicholas D. Allen, Julie Williams, Philip R. Taylor

**Affiliations:** ^1^ UK Dementia Research Institute at Cardiff Cardiff University Cardiff UK; ^2^ Systems Immunity Research Institute Cardiff University Cardiff UK; ^3^ School of Biosciences Cardiff University Cardiff UK

## Abstract

The Alzheimer's disease protective P522R *PLCG2* coding variant (rs72824905) is downstream of TREM2, but how it confers disease protection is poorly understood. Using a *Plcg2*‐R522 knock‐in mouse and *Plcg2*‐P522 control on both wildtype and Alzheimer's disease‐like *App* ^*NL‐G‐F*^ amyloidosis mouse backgrounds, aged mice were assayed for amyloid load, microglial activity, and synaptic integrity. In the absence of Alzheimer's disease‐like pathology, the R522 variant increased microglial coverage and was associated with reduced ramification complexity, fewer terminal points, and elevated lysosomal CD68 expression. On the *App* ^*NL‐G‐F*^ background, total amyloid burden was unaffected, but expression of the R522 variant led to increased plaque compaction compared to the P522 common variant. The protective R522 variant was also associated with: enhanced microglial engagement with less compact amyloid plaques; reduced microglial localisation around highly compacted plaques; protection from amyloid‐induced synapse loss; and decreased engulfment of synaptic material by microglia. Our data indicate a significant direct PLCγ2 role in controlling microglial‐plaque interactions and synaptic protection downstream of amyloid deposition, prioritizing it as a therapeutic target, potentially as an adjunct to other approaches, such as those targeting amyloid.

## Graphical abstract

The Alzheimer's disease protective PLCG2 P522R variant protects against amyloid‐induced synapse loss independently of amyloid load, promotes increased plaque compaction, and is associated with enhanced microglial engagement with diffuse amyloid.

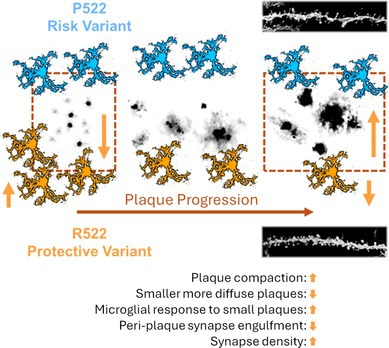

## 1 Introduction

A rare protective protein‐coding missense variant in the *PLCG2* gene (rs72824905, P522R) decreases the risk of developing late‐onset Alzheimer's disease (AD) (Sims et al. 2017). Human studies confirmed the relevance of the R522 variant with other dementias and associated it with increased longevity in centenarian cohorts (Bellenguez et al. 2022; Conway et al. 2018; van der Lee et al. 2019). This evidence supports a central role for *PLCG2* in neurodegenerative pathologies.

*PLCG2* encodes Phospholipase C Gamma 2 (PLCγ2), which, upon activation by specific membrane receptors or Rac GTPases, enables hydrolysis of phosphatidylinositol (van der Lee et al. 2019; Kadamur and Ross 2013) bisphosphate (PI(4,5)P2) into diacylglycerol (DAG) and inositol 1,4,5 trisphosphate (IP3). PLCγ2 activity modulates intracellular Ca^2+^ and protein kinase C signaling pathways (Kadamur and Ross 2013). In the periphery, PLCγ2 regulates various immune and biological processes, including B cell activation and function, peripheral inflammation, bone homeostasis, platelet activation, and the development of vasculature (Wang et al. 2001; Kurosaki et al. 2000; Ichise et al. 2009; Mao et al. 2006; Elvers et al. 2010). Notably, strong hypermorphic activity of PLCγ2, induced through gain‐of‐function variants such as S707Y (rs397514562), is implicated in rare immune disorders such as in the pathogenesis of APLAID (autoinflammation and PLCγ2‐associated antibody deficiency and immune dysregulation), highlighting its significance in immune regulation and autoinflammation (Zhou et al. 2012). In the brain, *PLCG2* is predominantly microglial expressed, positioning PLCγ2 within the same interaction network as *TREM2—*a notable AD risk gene (Andreone et al. 2020; Jonsson et al. 2013). Microglia are important for maintaining synaptic health, phagocytosis of debris, and secretion of inflammatory mediators, and are implicated in AD pathology (Paolicelli et al. 2011; Schafer et al. 2012; Keren‐Shaul et al. 2017).

Recent progress in AD therapeutics has seen some successes by promoting the removal of amyloid with monoclonal antibodies (Sims et al. 2023; Mintun et al. 2021; Van Dyck et al. 2023). These interventions have demonstrated a modest capacity to reduce cognitive decline in select patient subgroups, but are associated with the risk of side effects, including brain swelling and microhemorrhages. This underscores the necessity for identification of alternative or complementary therapeutic targets. The central role of PLCγ2 in modulating microglia and macrophage function, coupled with its inherent “druggability,” demands a deeper understanding of its contribution to disease mechanisms for its development as a therapeutic target.

We previously demonstrated that the PLCγ2‐R522 variant is hyperfunctional and alters microglial endocytic and phagocytic activity, including modestly increased β‐amyloid uptake in human iPSC‐derived microglia (Maguire et al. 2021). In AD mice, *Plcg2* is highly expressed in plaque‐associated microglia, increasing in abundance along the pathological timeline (Tsai et al. 2022; Magno et al. 2019). An initial study in a small cohort of *Plcg2* ^*R522*^ knock‐in mice reported enhanced microglial activity (Takalo et al. 2020). In a study of the 5xFAD model, the R522 variant reduced plaque load, and this was associated with improved downstream measures of behavior and synaptic function (Tsai et al. 2023). While we and others have observed clear impacts of the R522 variant on microglial function in vitro, its in vivo role in protection from critical disease‐relevant pathologies, such as synapse loss, is unknown and difficult to separate from any influence over amyloid burden.

Here, we explored the impact of the protective PLCγ2‐R522 variant on microglia‐synaptic homeostasis in situ in the context of the *App* ^*NL‐G‐F*^ knock‐in amyloid model. In spite of substantial amyloid burden, expression of the R522 variant resulted in enhanced plaque compaction and a marked protection of synaptic integrity, reinforcing the concept of PLCγ2 modulation as a neuropathology‐protective strategy.

## 2 Materials and Methods

### 2.1 Animal Models

All experiments were approved by the Animal Welfare and Ethical Review Body—subgroup of the Biological Standards Committee—and conducted in accordance with UK Home Office Guidelines and Animal [Scientific Procedures] Act 1986, which encompasses EU Directive 2010/63/EU on the protection of animals used for scientific purposes. Mouse models were based on the B6.Cg‐*Plcg2* ^em1Msasn^/J (*Plcg2* ^R522^) mice, available from The Jackson Laboratory (strain: 029598) (Maguire et al. 2021). Control mice were obtained from littermate founders (*Plcg2* ^P522^). Both *Plcg2* genotypes were crossed to the knock‐in *App* ^NL‐G‐F/NL‐G‐F^ mice to model amyloid‐driven pathology (Saito et al. 2014). Mouse genotypes were determined using real‐time PCR (Transnetyx, Cordova, TN). All mice were maintained and used as homozygous animals and cohoused in temperature‐controlled, pathogen‐free conditions with a 12‐h light/dark cycle and ad libitum access to food and water. Both male and female mice were included in all experiments. All mice were aged to 6 months for this study.

### 2.2 Brain Extraction and Preparation

Mice were euthanized by intraperitoneal administration of Euthatal (Merial Animal Health Ltd). After confirmation of death, brains were removed after transcardial perfusion with phosphate‐buffered saline (DPBS, Gibco, 14190136) and fixed in 1.5% paraformaldehyde (PFA) (Sigma, 1.00496) for > 72 h. Fixed brains were stored in DPBS containing 0.1% Sodium Azide (Sigma, S2002) at 4°C. Subsequently, free‐floating 50 μm coronal sections were created with a Leica VT1200S vibratome (Leica Biosystems). Coronal sections used were taken between −1.34 and −2.18 mm from the bregma.

### 2.3 Immunofluorescence

Following 30 min of heat‐induced epitope retrieval (citrate, Abcam, ab93678) sections were permeabilized with 1% Triton X‐100 (Merck, X100) for 10 min. After three washes in 0.05% Tween‐20 in DPBS (Promega, H5152), sections were blocked for 1 h at room temperature with 5% species‐specific normal sera (ThermoFisher) in 0.05% Tween‐20 in DPBS on an orbital shaker. Sections were then incubated at 4°C for 48 h with primary antibodies diluted in blocking buffer: rabbit anti‐Iba1 (1:2000, FUJIFILM Wako, 019‐19741), chicken anti‐Iba1 (1:250, Synaptic Systems, 234009), guinea pig anti‐Tmem119 (1:2000, Synaptic Systems, 400004), rat anti‐Clec7a (1:500, InvivoGen, mabg‐mdect‐2), rat anti‐CD68 (1:500, Bio‐Rad, MCA1957), rabbit anti‐PSD95 (1:500, Abcam, ab18258), guinea pig anti‐Bassoon (1:500, Synaptic Systems, 141004), guinea pig anti‐VGLUT2 (1:500, Synaptic Systems, 135404). After three washes in DPBS the samples were incubated with the appropriate species‐specific Alexa Fluor secondary antibodies (1:500, Invitrogen, A32740, A11006, A32759, A32740, A11073) in blocking buffer for 2 h at room temperature on an orbital shaker.

For antibody detection of amyloid plaques, brain sections were initially incubated in 70% formic acid (Merck, F0507) for 15 min at room temperature, followed by rehydration in water and DPBS for 20 min on a shaker. Sections were blocked as described above, before incubation for 72 h with anti‐Aβ fluorescent conjugated antibodies 6E10‐Alexa Fluor 594 (1:250, BioLegend, 803019) and 4G8‐Alexa Fluor 488 (1:250, BioLegend, 800714) in blocking buffer.

Labeled sections were counterstained with DAPI (Invitrogen, D1306) as required, and endogenous autofluorescence was quenched using 0.1% Sudan Black B (Sigma, 199664) in 70% ethanol, or for synaptic puncta related assays and measures from far‐red fluorophores, with TrueBlack Lipofuscin Autofluorescence Quencher (Biotium, 23007) according to manufacturer instructions. Isotype controls (Abcam and Invitrogen) and secondary‐only controls were used during the optimization process to ensure signal specificity.

Where necessary, amyloid plaques were stained using (1) Amylo‐Glo (Biosensis, TR‐300‐AG) according to manufacturer instructions, (2) Thioflavin S (ThioS) (0.1% (w/v), Sigma, T1892) dissolved in 70% ethanol and incubated for 20 min at room temperature, followed by three washes in 50% ethanol and rehydration in water and DPBS, or (3) X34 (Sigma, SML1954‐5MG), a fluorescent Congo Red derivative that binds β‐sheet secondary protein structures. For X34 staining, brain sections were incubated before blocking with 10 μM X34 diluted in 40% ethanol (vol/vol) in DPBS containing 20 mM NaOH for 20 min at room temperature (Baligács et al. 2024). The sections were then washed for 5 min in 40% ethanol (vol/vol) in DPBS with 20 mM NaOH, followed by rehydration in water and PBS. Where appropriate, sections were then stained with antibodies. All stained samples were mounted using VECTASHIELD Vibrance Antifade Mounting Medium (Vector, H‐1700), stored at 4°C in the dark, and imaged within 1–2 weeks.

### 2.4 Immunofluorescence and Plaque Image Acquisition and Analysis

Imaging was performed using a Leica SP8 Lightning confocal microscope with Leica HyD photodetectors set with a minimum 10% gain and fixed laser powers and a consistent scan speed of 600 Hz for each experimental assay. Precise settings used to image microglia, plaques, and synaptic puncta and the subsequent individual Imaris (v10.0, Bitplane) experiment analysis protocols are detailed in Supporting Information Methods.

### 2.5 DiOlistic Spine Labelling and Image Acquisition and Analysis

Free‐floating sections underwent DiOlistic labelling of hippocampal dendritic spines. DiOlistics was performed as previously described (Gan et al. 2000; Carpanini et al. 2022; Bevan et al. 2020). Dendritic spines labeled by DiOlistics were imaged from CA1 secondary dendrites within the stratum radiatum of the hippocampus and were selected based on minimal overlap with adjacent cells. The Leica SP8 Lightning confocal microscope was configured as detailed in Supporting Information Methods. Images were subsequently deconvolved using Leica Lightning Deconvolution software to resolve spine morphologies.

Dendritic spines were analyzed using the Filament Tracer function in Imaris (v9.9, Bitplane). “ROIs” nonoverlapping areas free from dye debris and other labelling, were positioned along dendrites greater than 30 μm in length, totalling 10 per mouse, and spines were defined as detailed in Supporting Information Methods.

### 2.6 Statistical Analysis

Data were analyzed, blinded to genotype and sex, with each experiment (i.e., one experimental marker/readout) processed in a single batch by the same operator. All graphs and statistical analyses were generated using GraphPad Prism (GraphPad Software, version 10), apart from mixed model analysis that was performed using RStudio (version 4.3.2). The Shapiro–Wilk test was used to check for normal distribution in all datasets with data log‐transformed if not normally distributed. Data were analyzed unless stated, by three‐way and two‐way ANOVA tests as applicable (considering genotype and sex) and, where relevant post hoc multiple comparisons (Bonferroni or two‐stage step‐up method for controlling false discovery rate [FDR]).

For 6E10/4G8 plaque analysis, the lmerTest package for mixed models in R was used to investigate the effects of the *Plcg2* variant and sex in each brain region, controlling for potential confounding variables for replicate and mouse variability. This was followed by an ANOVA (stats package—Type III Analysis of Variance Table with Satterthwaite's method) on the model output to determine the main and interaction effects. Significant interactions prompted multiple comparisons via the emmeans package, applying the Kenward–Rogers method for degrees of freedom and Tukey method for *p*‐value adjustment. Morphometric characteristics of the 6E10/4G8 plaques, including quantitative measures of plaque load, core status, quantity and sizes were “normalized” and scaled for dimensionality reduction using principal component analysis (PCA). Plaque cores (4G8) were aligned with their corresponding 6E10‐stained plaques, and X34‐stained plaques were paired with their respective peri‐plaque microglia for statistical analysis using Euclidean distance from relative Imaris position coordinates.

All data are presented as a mean value for individual mice, and group data generated from *n* = 10 males and 10 females from all genotypes is represented as mean ± standard deviation (SD), apart from Sholl analysis, which is presented as mean ± standard error of the mean (SEM). Statistical significance was set at *p* < 0.05 for all tests.

## 3 Results

### 3.1 Impact of the PLCγ2^R522^ Variant on the Microglial Network

**FIGURE 1:**
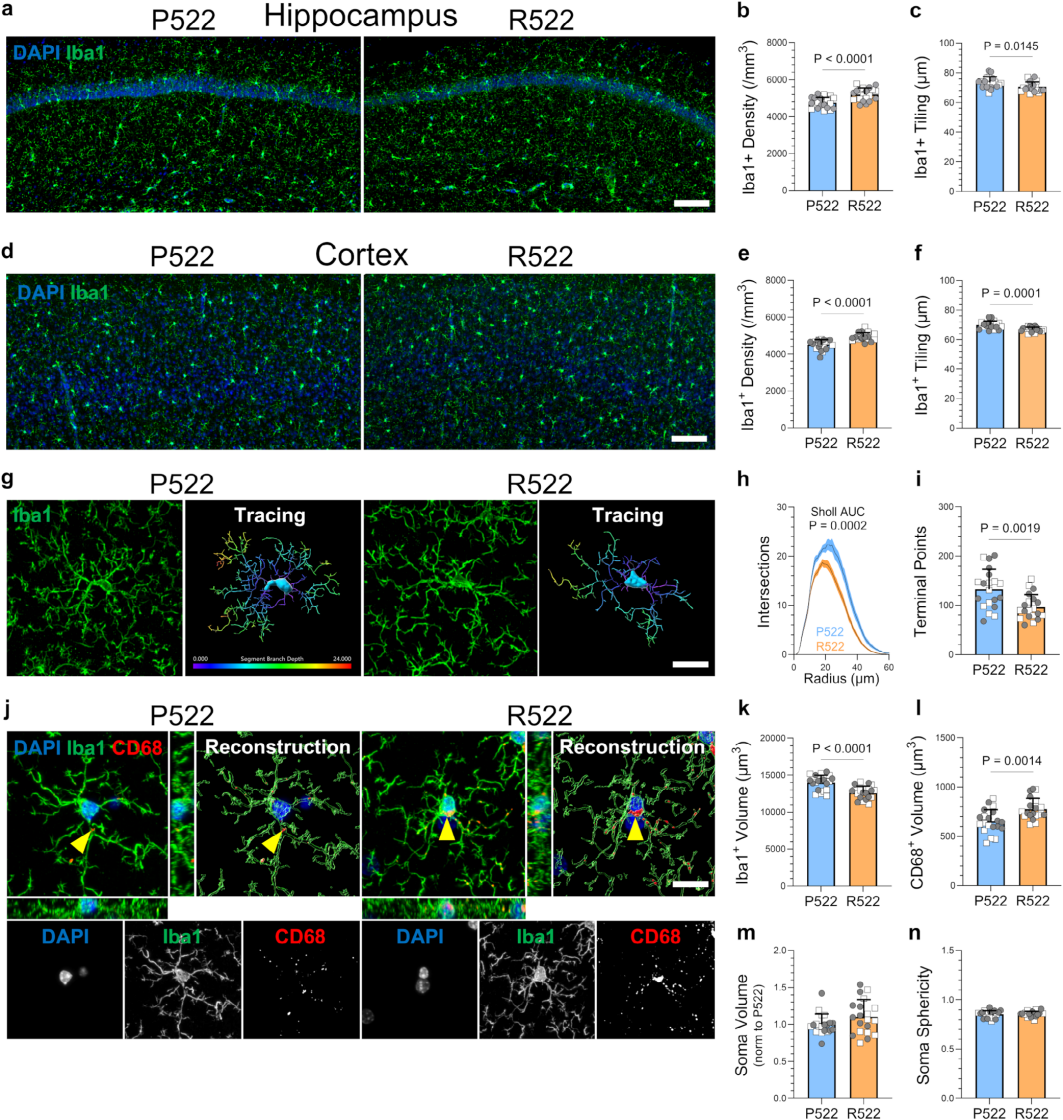
*Plcg2* ^*R522*^ variant induces changes in microglia density and morphology in the hippocampus and cortex of adult wildtype mice. (a) Representative image of microglia staining (Iba1, green) in the hippocampal CA1 region of wildtype (*Plcg2* ^*P522*^) and *Plcg2* ^*R522*^ variant mice. Scale bar: 100 μm. (b) Quantification of Iba1^+^ microglial cell density in the hippocampal CA1 region. (c) Microglial tiling score in the hippocampal CA1 region, measured as the average distance to the three nearest Iba1^+^ microglia. (d) Representative images of microglia staining (Iba1, green) in the cortex of wildtype (*Plcg2* ^*P522*^) and *Plcg2* ^*R522*^ variant mice. Scale bar: 100 μm. (e) Quantification of Iba1^+^ microglial cell density in the cortex. (f) Microglial tiling score in the cortex, measured as the average distance to the three nearest Iba1^+^ microglia. (g) Representative 3D images of Iba1^+^ microglial morphology in the hippocampal CA1 region, with accompanying 3D traced reconstructions showing soma (cyan) and branch depth. Scale bar: 30 μm. (h) Sholl analysis of Iba1^+^ microglia, reported as changes to the area under the curve (AUC). (i) Quantification of terminal points in Iba1^+^ microglia. (j) Representative 3D images of CD68^+^ lysosomal staining (red, yellow arrowheads) in Iba1^+^ microglia (green), with XZ and YZ views and corresponding 3D reconstruction. Scale bar: 30 μm. (k) Quantification of microglial Iba1^+^ cell volume. (l) Quantification of CD68^+^ puncta localized within Iba1^+^ microglia. (m, n) Measurements of microglial soma volume and soma sphericity in the hippocampus. All data points represent individual mice (*n* = 10 males [squares] and 10 females [circles] per genotype). In all graphs, data are summarized as mean ± SD, except for h, which is presented as mean ± SEM. (b, c) and (e, f) Averages from three entire fields of view per region (hippocampal CA1 stratum radiatum or cortex) within a 2.40 mm^2^ area (×10 objective). (h, i) and (k–n) Averaged from 10 3D images of microglia per mouse (×40 objective for (g), and ×63 objective for (j)). Statistical analysis was performed using two‐way ANOVA, accounting for genotype and sex, with no significant sex differences detected in these datasets. Shown *p*‐values reflect a significant effect of genotype (*Plcg2^R522^*): (b) interaction *p* = 0.4041; sex *p* = 0.4929; genotype *p* < 0.0001. (c) Interaction *p* = 0.8990; sex *p* = 0.9064; genotype *p* = 0.0145. (e) Interaction *p* = 0.9367; sex *p* = 0.2798; genotype *p* < 0.0001. (f) Interaction *p* = 0.5629; sex *p* = 0.9581; genotype *p* = 0.0001. (h) Interaction *p* = 0.4511; sex *p* = 0.5974; genotype *p* = 0.0002. (i) Interaction *p* = 0.5354; sex *p* = 0.6255; genotype *p* = 0.0019. (k) Interaction *p* = 0.2282; sex *p* = 0.3709; genotype *p* < 0.0001. (l) Interaction *p* = 0.8956; sex *p* = 0.1325; genotype = 0.0014. (m) Interaction *p* = 0.5483; sex *p* = 0.6648; genotype *p* = 0.1066. (n) Interaction *p* = 0.5381; sex *p* = 0.5187; genotype *p* = 0.7083.

To investigate the impact of the P522R variant on microglial morphology and spatial organization in vivo, we analyzed healthy 6‐month‐old mice expressing either the *Plcg2* ^*R522*^ or *Plcg2* ^*P522*^ variant (Figure 1). Mice expressing *Plcg2* ^*R522*^ exhibited a modest, but significantly higher density and tiling of Iba1^+^ microglia within the dorsal hippocampal CA1 region (Figure 1a–c) and the overlying cortices (Figure 1d–f) than *Plcg2* ^*P522*^ expressing mice. Sex had no detectable effect on these *Plcg2* variant‐related differences.

Microglial morphological analysis (CA1 region) revealed association of the *Plcg2* ^*R522*^ variant with lower ramification complexity (Figure 1g,h) and fewer terminal points (Figure 1i), consistent with a less arborized microglial phenotype. These morphological changes accompanied a significant reduction in microglial volume and increased prevalence of the lysosomal protein CD68, an indicator of phagocytic activity, in *Plcg2* ^*R522*^‐expressing microglia compared to those expressing *Plcg2* ^*P522*^ (Figure 1j–l). In contrast, measurements of cell soma volume and sphericity remained similar between the two genotypes (Figure 1m,n).

### 3.2 Altered Amyloid‐Plaque Pathology in the *App* ^*NL*^ ^*‐G‐F*^ Mouse With PLCγ2^R522^

**FIGURE 2:**
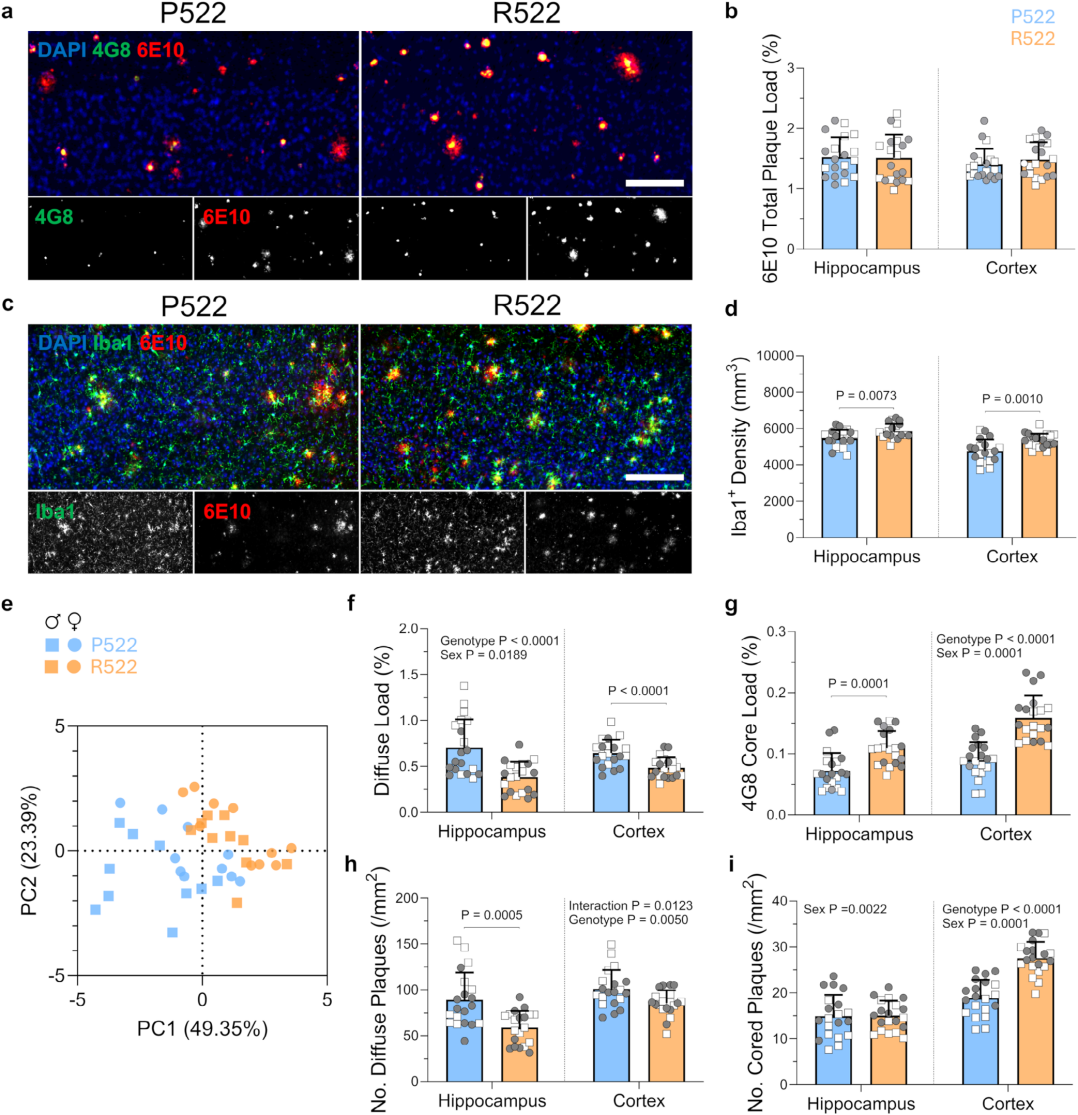
Altered amyloid burden associated with *Plcg2* ^*R522*^ variant expression in *App* ^*NL‐G‐F*^ mice. (a) Representative images of 6E10‐labeled plaques (red) and associated 4G8^+^ plaque cores (green) from the cortex *App* ^*NL‐G‐F*^ mice expressing either *Plcg2* ^*P522*^ or *Plcg2* ^*R522*^ variant. Scale bar: 100 μm. (b) Quantification of 6E10 total plaque load in the hippocampus CA1 and overlaying cortex. (c) Representative images of 6E10‐labeled plaques (red) costained with Iba1^+^ microglia (green) from the cortex *App* ^*NL‐G‐F*^ mice expressing either *Plcg2* ^*P522*^ or *Plcg2* ^*R522*^ variant. Scale bar 100 μm. (d) Iba1^+^ cell density in the hippocampus CA1 and overlying cortex. (e) Principal component analysis (PCA) of multiple plaque morphological and deposition parameters corresponding to the load, number and presence/absence of plaque cores. (f) Quantification of diffuse plaque load. (g) Quantification of plaque cores load (4G8^+^). (h) Number of diffuse plaques. (i) Number of 6E10 plaques with a core. All data points represent individual mice (*n* = 10 males [squares] and 10 females [circles] per genotype). In all graphs, data are summarized as mean ± SD. Data are presented as the average of three entire CA1 hippocampal or cortex fields, each within a 2.40 mm^2^ field of view (×10 objective). Statistical analysis was performed using linear mixed‐effects models for each region accounting for genotype and sex, for (b) and (d). (b) Hippocampus: interaction *p* = 0.9785, sex *p* = 0.5172, genotype *p* = 0.9012; cortex: interaction *p* = 0.6218, sex *p* = 0.5196, genotype *p* = 0.3641. (d) Hippocampus: interaction *p* = 0.8885, sex *p* = 0.1080, genotype *p* = 0.0073, cortex: interaction *p* = 0.1962, sex *p* = 0.0864, genotype *p* = 0.0010.

We introduced the R522 *Plcg2* variant into the *App* ^*NL‐G‐F*^ mouse model to evaluate its impact on amyloid‐driven pathology. By 6 months of age, *App* ^*NL‐G‐F*^ mice exhibit a substantial amyloid plaque burden in the brain (Saito et al. 2014). To characterize the plaques, two amyloid‐targeting antibodies, 6E10 and 4G8, were used (Baghallab et al. 2018). In *App* ^*NL‐G‐F*^ mice at 6 months of age, 6E10 labeled the majority of amyloid, including diffuse deposits, whereas 4G8 primarily marked the plaque cores (Figures 2a and S1a).

Quantification of the total 6E10^+^ plaque burden in the hippocampal CA1 region and the overlying cortex revealed no significant differences in amyloid pathology between mice expressing either *Plcg2* variant (Figures 2b and S1a). Next, we assessed microglial density in both brain regions using Iba1 as a pan‐microglial marker (Figures 2c and S1). Mice expressing the *Plcg2* ^*R522*^ variant exhibited a significantly increased microglial density in both the hippocampal CA1 region and the overlying cortex compared to the *Plcg2* ^*P522*^ mice (Figures 2d and S1b).

We characterized amyloid plaques based on their relative load, number, and classification as either diffuse or cored (based on absence or presence of a 4G8^+^ core, respectively). PCA revealed a clear distinction between *Plcg2* ^*P522*^ and *Plcg2* ^*R522*^ genotypes based on these parameters, with PC1 driven by global diffuse plaque burden (Figure 2e).

Quantification indicated a significant reduction in diffuse amyloid load in both the hippocampus and cortex of mice expressing the protective *Plcg2* ^*R522*^ variant compared to the common risk variant (Figure 2f). A sex effect was also observed, with males exhibiting a higher diffuse plaque burden in the hippocampus than females. Further analysis of 4G8^+^ core burden revealed that the protective *Plcg2* ^*R522*^ variant increased the coverage of plaque cores in both the hippocampus and cortex compared to the common risk variant, with a sex effect in the cortex associated with a higher plaque core burden in females than males (Figure 2g).

When assessing plaque number, we observed a reduction in diffuse plaques in *Plcg2* ^*R522*^‐expressing mice (Figure 2h). In the cortex, this reduction was primarily observed in male mice. Additionally, quantification of the number of cored plaques showed a significant increase in *Plcg2* ^*R522*^‐expressing mice, with an overall sex effect reflecting a higher number of cored plaques in females in both brain regions (Figure 2i).

To support these findings, we used Thioflavin S (ThioS) staining, which labels fibrillar amyloid, to examine plaque coverage in the hippocampus and cortex (Figure S2). In the hippocampus, ThioS staining revealed an increased area of coverage associated with a greater number of individual plaques, larger plaques, and more densely compacted plaques in *Plcg2* ^*R522*^‐expressing mice compared to P522 carriers (Figure S2a–e). In the cortex, female *App* ^*NL‐G‐F*^ mice exhibited a higher ThioS‐labeled plaque burden than males (Figure S2f,g). Notably, in male *App* ^*NL‐G‐F*^ mice, the R522 variant was associated with an increase of ThioS^+^ plaques in the cortex (Figure S2g–j). Although overall ThioS plaque load in female *Plcg2* ^*R522*^ mice remained unchanged, these plaques were more densely compacted than those in P522 carriers (Figure S2g–j).

### 3.3 PLCγ2^R522^ Alters Amyloid‐Plaque Compaction in the *App* ^*NL*^ ^*‐G‐F*^ Mouse

Since the initial data indicated differences in plaque morphometrics in the *Plcg2* ^*R522*^‐expressing *App* ^*NL‐G‐F*^ mice, we analyzed plaques in the hippocampus CA1 region based on their size and compactness (see Supporting Information) (Preman et al. 2024). Plaques were characterized using X34, a dye that strongly binds β‐sheeted amyloid, labelling both dense plaque cores and surrounding amyloid deposits, albeit with lower intensity in the latter (Baligács et al. 2024).

**FIGURE 3:**
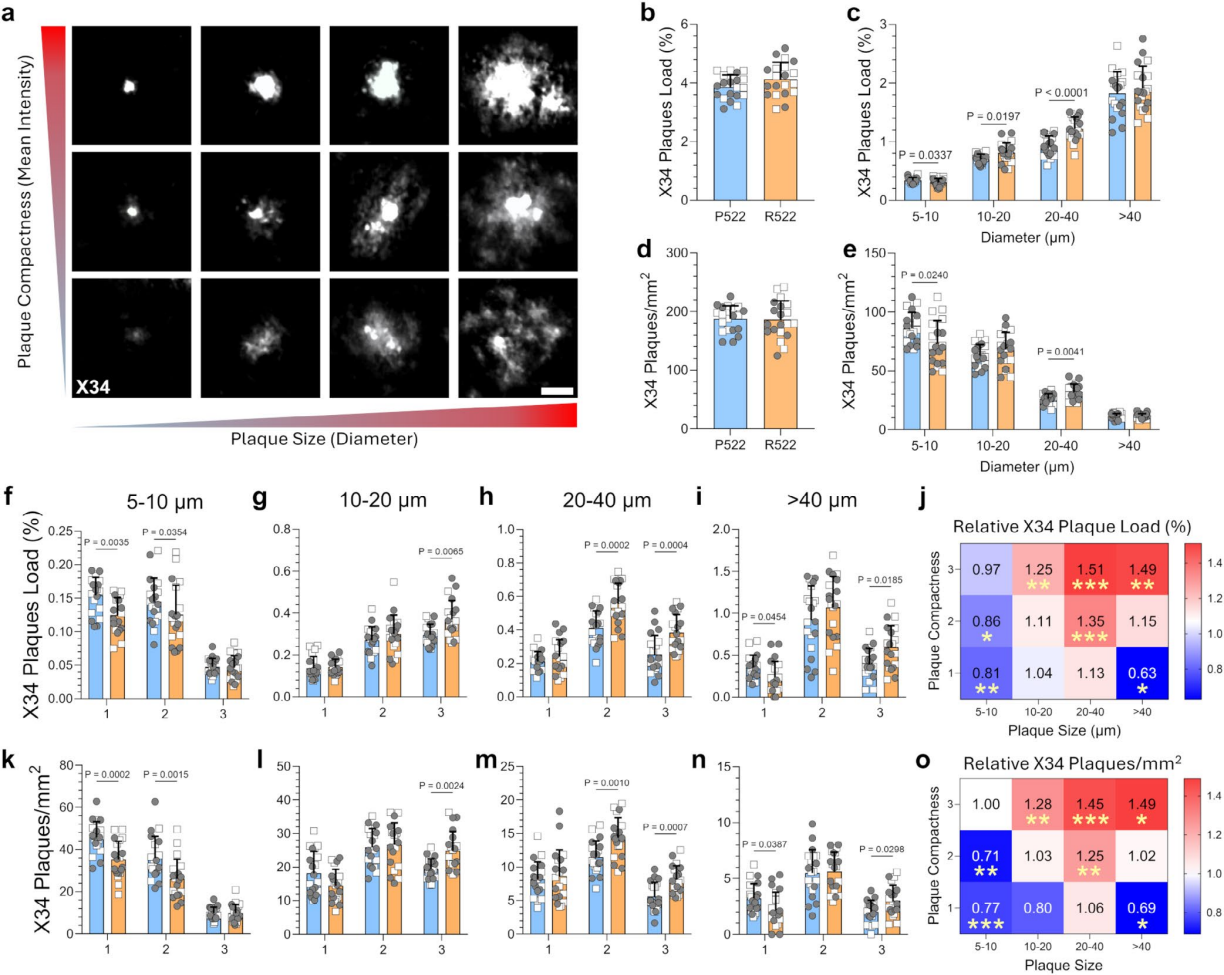
*Plcg2* ^*R522*^ variant drives plaque compaction in *App* ^*NL‐G‐F*^ mice. (a) Representative images from the hippocampal CA1 regions of X34‐labeled plaques (white) categorized based on plaque size (5–10, 10–20, 20–40, or > 40 μm) and plaque compactness (diffuse, cored with surrounding diffuse amyloid, cored compact plaques). Scale bar 20 μm. (b) X34 plaque load. (c) X34 plaque load based on plaque diameter. (d) Number of X34 plaques. (e) Number of X34 plaques based on plaque diameter. (f–i) X34 plaque load relative to the plaque diameters and level of compactness (1, diffuse; 2, cored with surrounding diffuse amyloid; 3, cored compact). (j) Heatmap summary representing the fold change between genotypes for each plaque categorization for X34 plaque load. (k–n) Number of X34 plaques relative to the plaque diameters and level of compactness (1, diffuse; 2, cored with surrounding diffuse; 3, cored compact). (o) Heatmap summary representing the fold change between genotypes for each plaque categorization for the number of X34 plaques. All data points represent individual mice (*n* = 10 males [squares] and 10 females [circles] per genotype). In all graphs, data are summarized as mean ± SD. Plaque data obtained from three CA1 hippocampal fields (×20 objective). Statistical analysis for (f–o) used three‐way ANOVA per plaque categorization (genotype, plaque compactness, sex). If no sex effects were found, data were pooled and analyzed with two‐way ANOVA (genotype, plaque compactness). *p* Values were reported correspond to two‐stage step‐up method controlling for false discovery rate (FDR). For (b, d), two‐way ANOVA was used per region (genotype, sex). For (c, e), three‐way ANOVA was used per plaque categorization (genotype, plaque diameter, sex), with data pooled for two‐way ANOVA (genotype, plaque diameter) if no sex effects were detected. (b) Interaction *p* = 0.3510, sex *p* = 0.7856, genotype *p* = 0.1076. (C) Interaction *p* = 0.0082, plaque diameter *p* < 0.0001, genotype *p* = 0.0055. (d) Interaction *p* = 0.7992, sex *p* = 0.2428, genotype *p* = 0.8967. (e) Interaction *p* = 0.0001, plaque diameter *p* < 0.0001, genotype *p* = 0.9104. (f) Interaction *p* = 0.0705, plaque compactness *p* < 0.0001, genotype *p* = 0.0063. (g) Interaction *p* = 0.0783, plaque compactness *p* < 0.0001, genotype *p* = 0.0053. (h) Interaction *p* = 0.0404, plaque compactness *p* < 0.0001, genotype *p* < 0.0001. (i) Interaction *p* = 0.0351, plaque compactness *p* < 0.0001, genotype *p* = 0.1349. (k) Interaction *p* = 0.0028, plaque compactness *p* < 0.0001, genotype *p* = 0.0001. (l) Interaction *p* = 0.0013, plaque compactness *p* < 0.0001, genotype *p* = 0.4496. (m) Interaction *p* = 0.1235, plaque compactness *p* < 0.0001, genotype *p* = 0.0004. (n) Interaction *p* = 0.0195, plaque compactness *p* < 0.0001, genotype *p* = 0.8755. *p < 0.05, **p < 0.01, ***p < 0.001, ****p < 0.0001.

Plaques were categorized by diameter (5–10, 10–20, 20–40, or > 40 μm) and compactness (on an increasing scale of 1–3), measured using X34 mean intensity. This classification distinguished diffuse/low‐compactness plaques, intermediate compact plaques with surrounding diffuse amyloid, and highly compacted core‐dominant plaques, forming a 3 × 4 grid classification system (Figure 3a). We then assessed overall X34 load and plaque count, as well as these metrics within each plaque category.

There was no significant difference in overall X34 plaque load between mice expressing the protective *Plcg2* ^*R522*^ variant and those expressing the common risk variant (Figure 3b). However, when stratifying plaques by diameter (Figure 3c), *Plcg2* ^*R522*^‐expressing mice exhibited a reduced plaque load in the 5–10 μm range but an increased load in the 10–20 and 20–40 μm groups, with no differences observed in plaques > 40 μm. Similarly, total X34 plaque count did not differ significantly between genotypes (Figure 3d). However, when stratified by diameter (Figure 3e), *Plcg2* ^*R522*^‐expressing mice exhibited fewer 5–10 μm plaques than *Plcg2* ^*P522*^‐expressing mice but an increased number in the 20–40 μm plaques, with no differences observed with the 10–20 or > 40 μm sizes.

We measured X34 load and count for each plaque category, restricted within their respective diameter ranges and plaque compactness, to assess plaque distribution in the *App* ^*NL‐G‐F*^ brain and the influence of the *Plcg2* genotype. The *Plcg2* ^*R522*^ variant decreased both the load and number of 5–10 μm plaques with low compactness (Levels 1 and 2) (Figure 3f,k). For 10–20 μm plaques, the *Plcg2* ^*R522*^ protective variant increased both load and count of the most compact plaques (Level 3) (Figure 3g,l). In the 20–40 μm range, *Plcg2* ^*R522*^‐expressing mice exhibited elevated plaque load and number for compactness Levels 2 and 3 (Figure 3h,m). For plaques > 40 μm, the protective variant decreased both measures for compactness Level 1 but increased them for plaques scored at compactness Level 3 (Figure 3i,n). Representing these datasets as fold change between the variants for each plaque category (Figure 3j,o) visualizes a shift in plaque morphology in *Plcg2* ^*R522*^‐expressing mice, with reduced coverage and number of smaller diffuse plaques and an increased coverage and numbers of larger compacted plaques.

### 3.4 Heightened Microgliosis to Diffuse Amyloid Pathology Associated With PLCγ2^R522^

**FIGURE 4:**
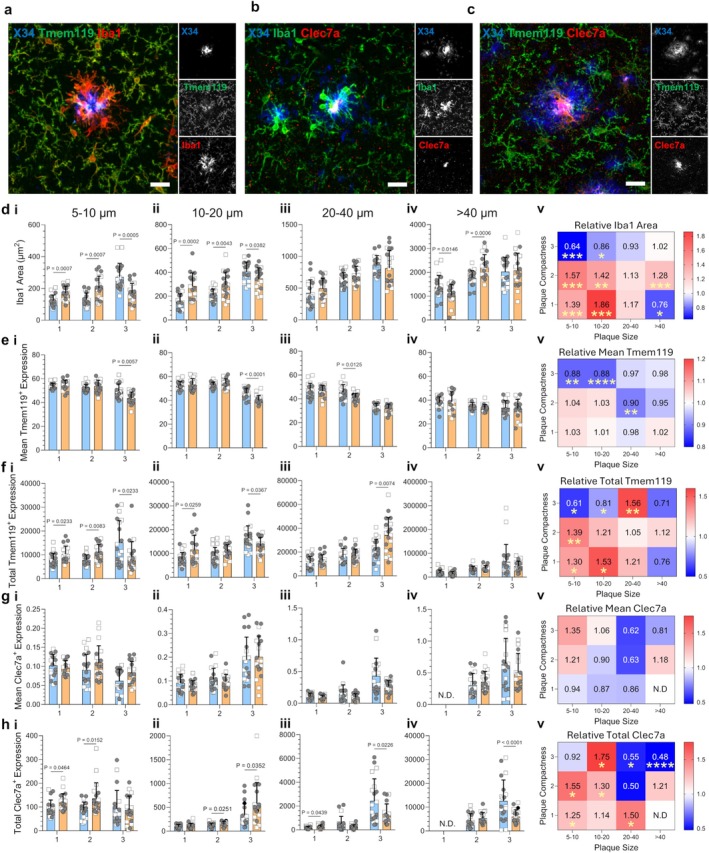
*Plcg2* ^*R522*^ variant influences peri‐plaque associated microglia in *App* ^*NL‐G‐F*^ mice. (a) Representative image of an X34‐labeled plaque in the hippocampal CA1 region, costained with Tmem119 and masked from Iba1 in an *App* ^*NL‐G‐F*^ mouse. (b) Representative image of an X34‐labeled plaque in the hippocampal CA1 region, costained with Clec7a and masked from Iba1 in an *App* ^*NL‐G‐F*^ mouse. (c) Representative image of an X34‐labeled plaque in the hippocampal CA1 region, costained with Tmem119 and Clec7a in an *App* ^*NL‐G‐F*^ mouse. (d, i–iv) Peri‐plaque Iba1 area response relative to the plaque diameters and level of compactness (1, diffuse; 2, cored with surrounding diffuse amyloid; 3, cored compact). (d, v) Heatmap summary representing the fold change between genotypes for each plaque categorization for peri‐plaque Iba1 Area. (e, i–iv) Peri‐plaque Mean Tmem119 microglial expression relative to the plaque diameters and level of compactness (1, diffuse; 2, cored with surrounding diffuse amyloid; 3, cored compact). (e, v) Heatmap summary representing the fold change between genotypes for each plaque categorization for peri‐plaque mean Tmem119 microglial expression. (f, i–iv) Peri‐plaque Total Tmem119 microglial expression relative to the plaque diameters and level of compactness (1, diffuse; 2, cored with surrounding diffuse amyloid; 3, cored compact). (f, v) Heatmap summary representing the fold change between genotypes for each plaque categorization for peri‐plaque Total Tmem119 microglial expression. (g, i–iv) Peri‐plaque Mean Clec7a microglial expression relative to the plaque diameters and level of compactness (1, diffuse; 2, cored with surrounding diffuse amyloid; 3, cored compact). (g, v) Heatmap summary representing the fold change between genotypes for each plaque categorization for peri‐plaque mean Clec7a microglial expression. (h, i–iv) Peri‐plaque Total Clec7a microglial expression relative to the plaque diameters and level of compactness (1, diffuse; 2, cored with surrounding diffuse amyloid; 3, cored compact). (h, v) Heatmap summary representing the fold change between genotypes for each plaque categorization for peri‐plaque Total Clec7a microglial expression. All data points represent individual mice (*n* = 10 males [squares] and 10 females [circles] per genotype). Insufficient *N* mice for v and ab plaque level compactness 1. In all graphs, data are summarized as mean ± SD. Plaque data obtained from three CA1 hippocampal fields (×20 objective). Statistical analysis used three‐way ANOVA per plaque categorization (genotype, plaque compactness, sex). If no sex effects were found, data were pooled and analyzed with two‐way ANOVA (genotype, plaque compactness). *p* Values were reported correspond to two‐stage step‐up method controlling for false discovery rate (FDR). (d, i) Interaction *p* < 0.0001, plaque compactness *p* < 0.0001, genotype *p* = 0.5444. (d, ii) Interaction *p* < 0.0001, plaque compactness *p* < 0.0001, genotype *p* = 0.0033. (d, iii) Interaction *p* = 0.0973, plaque compactness *p* < 0.0001, genotype *p* = 0.5449. (d, iv) Interaction *p* = 0.0023, plaque compactness *p* < 0.0001, genotype *p* = 0.4815. (e, i) Interaction *p* < 0.0001, plaque compactness *p* < 0.0001, genotype *p* = 0.2820. (e, ii) Interaction *p* < 0.0001, plaque compactness *p* < 0.0001, genotype *p* = 0.1884. (e, iii) Interaction *p* = 0.1073, plaque compactness *p* < 0.0001, genotype *p* = 0.0396. (e, iv) Interaction *p* = 0.6605, plaque compactness *p* < 0.0004, genotype *p* = 0.6322. (f, i) Interaction *p* < 0.0001, plaque compactness *p* = 0.0205, genotype *p* = 0.7830. (f, ii) Interaction *p* = 0.0007, plaque compactness *p* < 0.0001, genotype *p* = 0.2443. (f, iii) Interaction *p* = 0.0042, plaque compactness *p* < 0.0001, genotype *p* = 0.0034. (f, iv) Interaction *p* = 0.2914, plaque compactness *p* < 0.0001, genotype *p* = 0.2858. (g, i) Interaction *p* = 0.0936, plaque compactness *p* = 0.0006, genotype *p* = 0.1010. (g, ii) Interaction *p* = 0.6311, plaque compactness *p* < 0.0001, genotype *p* = 0.7818. (g, iii) Interaction *p* = 0.0399, plaque compactness *p* < 0.0001, genotype *p* = 0.0083. (g, iv) Interaction *p* = 0.0615, plaque compactness *p* < 0.0001, genotype *p* = 0.7382. (h, i) Interaction *p* = 0.0587, plaque compactness *p* = 0.2214, genotype *p* = 0.0445. (h, ii) Interaction *p* = 0.0245, plaque compactness *p* < 0.0001, genotype *p* = 0.0108. (h, iii) Interaction *p* = 0.0087, plaque compactness *p* < 0.0001, genotype *p* = 0.0148. (h, iv) Interaction *p* = 0.0010, plaque compactness *p* < 0.0001, genotype *p* = 0.0213.

We next assessed microglial responses at the peri‐plaque level in the *App* ^*NL‐G‐F*^ mice expressing either the protective *Plcg2* ^*R522*^ variant or the common risk variant. To investigate key aspects of this response, plaques were categorized by diameter and compactness (as above) and microglial activation assessed using the pan‐microglial marker Iba1, the homeostatic microglial marker Tmem119, and the disease‐associated microglia (DAM) marker Clec7a, costained with X34‐labeled amyloid plaques (Figure 4a–c).

Assessment of peri‐plaque microgliosis, measured as Iba1 area within 5 μm of plaque borders, indicated significant increases in microglia associated with 5–10 μm (Figure 4d,i) and 10–20 μm (Figure 4d,ii) plaques in the R522 variant‐expressing mice, but only those with lower compactness (Levels 1 and 2). Notably, the most compact plaques within these size ranges showed a strong reduction in microglial association (Figure 4d,i,ii). No significant changes in microgliosis were detected for any 20–40 μm plaques (Figure 4d,iii). However, in the largest plaques (> 40 μm), we observed a reduction in microgliosis at the lowest compactness level, an increase at compactness Level 2, and no changes in the most compact plaques (Figure 4d,iv). Representing these datasets as relative expression between the protective and common risk variants (Figure 4d,v) illustrated that, with regard to the more numerous smaller plaques, *Plcg2* ^*R522*^‐expressing mice exhibited an enhanced Iba1 microglial response toward lower‐compactness plaques, with a converse reduction in microglial association around the most compact plaques.

An Iba1^+^ peri‐plaque microglia mask was used to assess Tmem119 staining, and the Tmem119 signature was quantified in two ways: mean expression, representing the relative expression level per microglia, and total expression, indicating the overall Tmem119 microglial signal within the peri‐plaque region. Tmem119 expression per microglia remained similar between the two genotypes (Figure 4e,i–iv). However, in the *Plcg2* ^*R522*^‐expressing mice, a reduction in Tmem119^+^ expression per microglia was associated with the most compact 5–10 μm (Figure 4e,i) and 10–20 μm (Figure 4e,ii) plaques, as well as in 20–40 μm plaques at compactness Level 2 (Figure 4e,iii). When represented as fold change between the *Plcg2* genotypes (Figure 4e,v), a pattern toward reduced Tmem119 expression in microglia associated with smaller, more compact plaques was seen with *Plcg2* ^*R522*^‐expression.

Tmem119^+^ microgliosis in response to plaques generally mirrored the initial Iba1^+^ area response but displayed distinct differences. Total Tmem119 peri‐plaque expression for the smaller plaques (Figure 4f,i,ii) was broadly consistent with the general microglial distribution data (Figure 4d,v). For the most compact (Level 3) 20–40 μm plaques, an increase was detected in the *Plcg2* ^*R522*^‐expressing mice (Figure 4f,iii). No significant differences between the variants were observed for larger plaques (Figure 4f,iv). Visualized as fold change between the genotypes (Figure 4f,v), a pattern in *Plcg2* ^*R522*^‐expressing mice of heightened Tmem119^+^ microglia surrounding smaller, lower‐compactness plaques, with contrasting reduction around the most compact smaller plaques.

In contrast, mean Clec7a expression was similar between the protective and common risk variants across all plaque types (Figure 4g,i–iv). When assessed as total Clec7a expression, we observed changes, particularly, around smaller, lower‐compactness plaques, that reflected microglial density (Figure 4h,i,ii), similar to the pattern seen with total Tmem119 expression. Notable changes included increased presence of Clec7a expressing microglia in the most compact 10–20 μm plaques of *Plcg2* ^*R522*^ mice (Figure 4h,ii) in direct opposition of the reduced presence of Tmem119^+^ microglia seen (Figure 4f, ii). Conversely, the most compact 20–40 μm plaques exhibited lowest presence of Clec7a expressing microglia in *Plcg2* ^*R522*^ mice (Figure 4h,iii) contrasting with an increased presence of Tmem119 expression (Figure 4f,iii). This significant drop in Clec7a association was also evident in the largest most compact plaques (Figure 4h,iv). When visualized relative to the *Plcg2* ^*P522*^‐expressing mice (Figure 4h,v), a pattern toward elevated Clec7a expression in the *Plcg2* ^*R522*^‐expressing mice in response to smaller, lower‐compactness plaques, which was significantly reduced in the largest, most compacted plaques was observed. Global *Clec7a* expression, measured by qPCR in the cortex, revealed a substantial increase in *App* ^*NL‐G‐F*^ mice compared to wildtype, but no differences between *Plcg2* ^*R522*^‐ and *Plcg2* ^*P522*^‐expressing *App* ^*NL‐G‐F*^ mice. However, in wildtype mice, *Plcg2* ^*R522*^ was associated with increased *Clec7a* expression, highlighting the need for spatially resolved analyses to detect microglial differences in the amyloid models when looking at changes likely to have milder phenotypic effects (Figure S3a).

To further examine the inflammatory response, we assessed *Tnf* and *Il6* by qPCR in the cortex (Supporting Information Methods and Figure S3b). Six‐month‐old *App* ^*NL‐G‐F*^ mice expressing *Plcg2* ^*R522*^ exhibited significantly increased *Tnf* expression. Other in vivo studies of the *Plcg2* variants have proven very variable with no clear pattern (Tsai et al. 2023; Takalo et al. 2025). It has been previously reported that *Plcg2* ^*R522*^ expression may enhance the inflammatory response to a limited in vitro challenge (Takalo et al. 2020), hence we elected to conduct a time course analysis of cytokine responses to the model microbial challenge, LPS, or Aβ_1‐42_. We selected human induced pluripotent stem cell (iPSC)‐derived microglia as it allowed us to test the more directly relevant human cell response (Figure S3c) (Maguire et al. 2021). Upon stimulation with Aβ, a modest TNF response was elicited, which was significantly higher in *PLCG2* ^*R522*^ expressing cells and largely abrogated by deficiency of *PLCG2*. The G‐CSF response was fairly substantial, but markedly reduced in the R522 variant expressing cells, and again largely abrogated by *PLCG2* deficiency. With LPS as a stimulus, the TNF response was more substantial, but significantly lower in the *PLCG2* ^*R522*^ expressing cells, and similar to the *PLCG2*‐KO cells. In this case, the G‐CSF response to LPS was much more muted and not significantly affected by the *PLCG2* genotype. Instead, R522 expressing cells, similar to the *PLCG2*‐KO cells, exhibited a significantly reduced IL‐6 response to LPS compared to the P522 cells, mimicking the nonsignificant appearance of the IL‐6 response to Aβ.

### 3.5 PLCγ2^R522^ Protects Synaptic Integrity in *App* ^*NL*^ ^*‐G‐F*^ Mice

**FIGURE 5:**
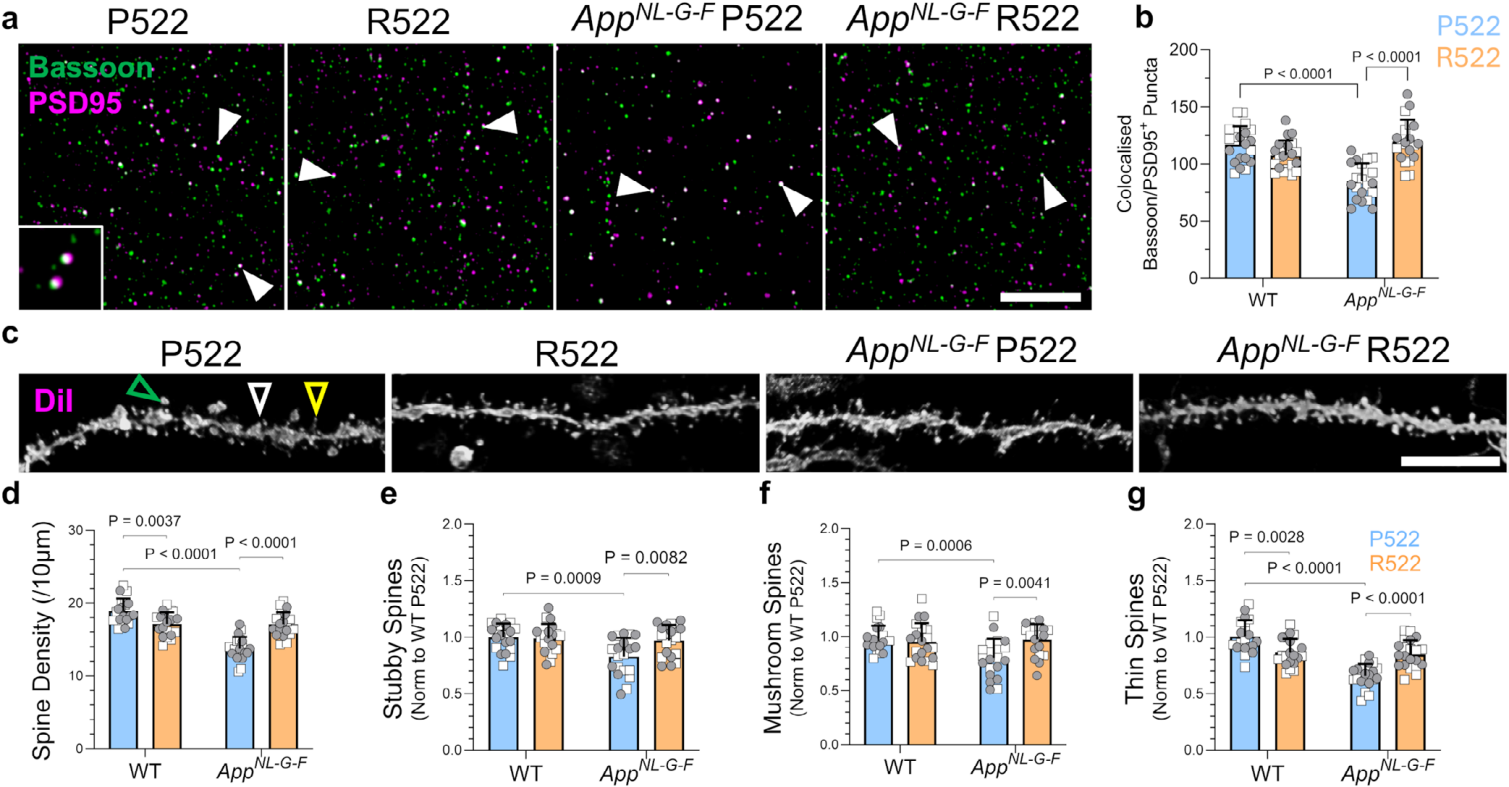
*Plcg2* ^*R522*^ expression protects synapses in *App* ^*NL‐G‐F*^ mice. (a) Hippocampal CA1 presynaptic bassoon (green) and postsynaptic PSD95 (magenta) synaptic puncta greater than 30 μm from plaque cores from wildtype and *App* ^*NL‐G‐F*^ mice with the risk P522 and protective R522 *Plcg2* variants. Insert and arrows indicate examples of colocalised bassoon and PSD95 (white). Scale bar 10 μm. (b) Quantification of hippocampal CA1 colocalised bassoon and PSD95 synaptic puncta greater than 30 μm from plaque cores in WT and *App* ^*NL‐G‐F*^ mice with the risk P522 and protective R522 *Plcg2* variants. (c) DiOlistic labeled hippocampal CA1 dendrites with spine protrusions. Empty arrowheads correspond to stubby (white), mushroom (green), and thin (yellow) spine subtypes. Scale bar 10 μm. (d) Overall spine densities. (e–g) Relative proportions of stubby, mushroom and thin spine subtypes (based on morphological head and neck parameters). All data points represent individual mice (*n* = 10 males [squares] and 10 females [circles] per genotype). (b) Data represented as the average of 6 (30 μm × 30 μm) ROI from the CA1 stratum radiatum hippocampal field per mouse. (d–g) Data represented as the average of 10 dendritic segments traced per mouse from the CA1 hippocampal apical secondary dendrites within the stratum radiatum. In all graphs, data are summarized as mean ± SD. Data were analyzed by Three‐way ANOVA considering *Plcg2* P522R variants, *App* ^*NL‐G‐F*^ status and sex for (b) and (d–g); no sex differences were detected for the hippocampus datasets and therefore the sex cofactor was consolidated for Two‐way ANOVA considering both *Plcg2* and *App* ^*NL‐G‐F*^ genotypes. All *p* value reported represents the post hoc multiple comparisons test (Bonferroni). (b) Interaction *p* < 0.0001, App *p* = 0.0073, Plcg2 *p* = 0.0008. (d) Interaction *p* < 0.0001, App *p* < 0.0001, Plcg2 *p* = 0.0369. (e) Interaction *p* = 0.0177, App *p* = 0.0031, Plcg2 *p* = 0.0399. (f) Interaction *p* = 0.0029, App *p* = 0.0137, Plcg2 *p* = 0.0753. (g) Interaction *p* < 0.0001, App *p* < 0.0001, Plcg2 *p* = 0.3304.

Next, we quantified synaptic puncta density in the hippocampal CA1 region of wildtype and *App* ^*NL‐G‐F*^ mice expressing either *Plcg2* genotype. Presynaptic (bassoon) and postsynaptic (PSD95) were assessed in regions > 30 μm from AmyloGlo^+^ plaque cores to measure synaptic density away from plaques (Figure 5a). In *App* ^*NL‐G‐F*^ mice, those expressing the *Plcg2* ^*P522*^ variant exhibited a significant reduction in colocalised synaptic puncta compared to wildtype controls, whereas *App* ^*NL‐G‐F*^ mice with the *Plcg2* ^*R522*^ variant showed no such loss (Figure 5b). In the absence of *App* ^*NL‐G‐F*^, there was no significant alteration in synaptic puncta density associated with *Plcg2* genotype (Figure 5b). We extended these studies to examine the hippocampus (Figure S4a,b,e,f) and cortex (Figure S4c,d,g,h) and also to divide the regions into peri‐plaque (Figure S4e–h) and distal from the plaque (Figure S4a–d) regions (Supporting Information Methods). These observations were consistent between the regions of the brain, with lowest colocalised puncta counts in the peri‐plaque region. The R522 variant was protective in both brain regions and independent of plaque proximity. Interestingly, a sex‐specific effect of the PLCγ2 variants was observed in the cortex of males distal from the plaques (Figure S4d). Further analysis showed higher peri‐plaque density of VGLUT2^+^ excitatory synapses in *Plcg2* ^*R522*^‐expressing *App* ^*NL‐G‐F*^ mice (Figure S4i,j).

Dendritic spine analysis, a key indicator of postsynaptic integrity in CA1 neurons, was conducted using an established labelling method (Figures 5c and S5) (Carpanini et al. 2022; Bevan et al. 2020). *App* ^*NL‐G‐F*^ mice displayed a marked reduction in dendritic spine density compared to wildtype controls; however, this loss was significantly attenuated in *Plcg2* ^*R522*^ expressing mice (Figure 5d). Spines were further categorized into stubby, mushroom, and thin subtypes (Figure 5e–g, Supporting Information Methods). In *App* ^*NL‐G‐F*^ mice with the *Plcg2* ^*P522*^ variant, spine loss was evident across all subtypes, whereas the *Plcg2* ^*R522*^ variant preserved spine density across each category, with values similar to *Plcg2* ^*R522*^‐expressing mice lacking *App* ^*NL‐G‐F*^ (Figure 5e–g). In wildtype mice, the *Plcg2* ^*R522*^ variant was associated with subtle synaptic remodeling, reflected by a modest decrease in total spine density (Figure 5d), associated with a reduction in thin spines (Figure 5g).

### 3.6 *Plcg2* ^*R522*^ Reduces Microglial Engulfment of Synapses at Plaques

**FIGURE 6:**
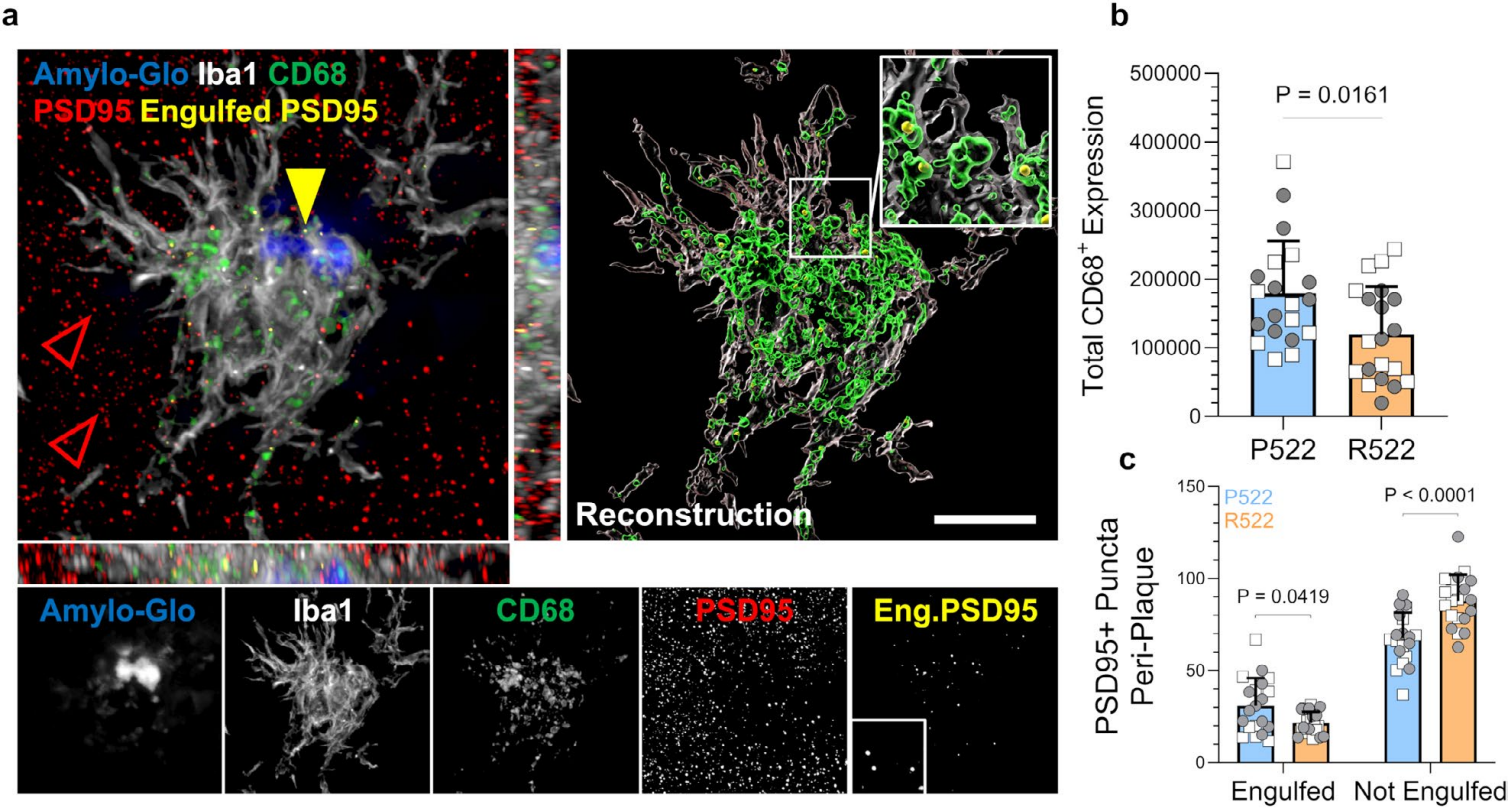
Plcg2^R522^ variant protects against synaptic engulfment at plaques in *App* ^*NL‐G‐F*^ mice. (a) Representative *App* ^*NL‐G‐F*^ example of 3D image of hippocampal CA1 plaque for analysis of peri‐plaque regions of interest (within 30 μm radius around plaque core) for synaptic engulfment in *App* ^*NL‐G‐F*^ mice with the risk P522 and protective R522 *Plcg2* variants. Amylo‐Glo (blue), Iba1 (white), CD68 (green), PSD95 (red, synaptic puncta), and engulfed PSD95 (PSD95, CD68‐colabelling, yellow). Yellow arrowheads indicate areas of engulfed PSD95 inside CD68 lysosomes, empty red arrowheads indicate not engulfed PSD95. Scale bar 5 μm. (b) CD68^+^ total expression peri‐plaque response. (c) Quantification of hippocampal CA1 PSD95 synaptic puncta peri‐plaque separated between engulfed (i.e., within CD68^+^ lysosomes) and not engulfed puncta surrounding the plaque. White arrowheads indicates areas pre‐ and postsynaptic colocalisation. Data represent an average of 5 3D plaque core images of similar size per mouse (×63 objective) from the CA1 stratum radiatum hippocampal field. All data points represent individual mice (*n* = 10 males [squares] and 10 females [circles] per genotype). In all graphs, data are summarized as mean ± SD. Data were analyzed by Two‐way ANOVA considering genotype and sex for (b), and three‐way ANOVA considering *Plcg2* P522R variants, engulfment and sex for (c); no sex differences were detected for the hippocampus datasets and therefore the sex cofactor was consolidated in for two‐way ANOVA considering *Plcg2* genotype. (b) Interaction *p* = 0.4844, Sex *p* = 0.9496, genotype *p* = 0.0161. (c) Interaction *p* < 0.0001, PSD95 puncta engulfed/not engulfed *p* < 0.0001, genotype *p* = 0.0679.

As the *Plcg2* ^*R522*^ variant confers a protective effect on synaptic integrity in *App* ^*NL‐G‐F*^ mice, we examined microglial synaptic engulfment within the peri‐plaque environment. AmyloGlo‐labeled plaques were co‐stained with Iba1, CD68, and PSD95 (Figure 6a). In *Plcg2* ^*R522*^‐expressing *App* ^*NL‐G‐F*^ mice, microglia exhibited a significantly reduced CD68‐to‐Iba1 ratio at peri‐plaque niches compared to those expressing Plcg2^P522^ (Figure 6b), which contrasted with observations in the absence of *App* ^*NL‐G‐F*^ (Figure 1j–l). Quantification of PSD95 puncta within these regions revealed a lower proportion of engulfed synapses and a higher prevalence of unengulfed PSD95^+^ synapses in Plcg2^R522^‐expressing mice relative to their Plcg2^P522^ counterparts (Figure 6c).

## 4 Discussion

Through study of the *App* ^*NL‐G‐F*^ mouse model, we have found that the R522 AD‐protective variant drives multiple pathological changes in the context of limited impact on the total amyloid burden. Specifically, the R522 variant manifests: (i) a skewing of plaque morphology toward larger, more compact plaques and away from smaller, diffuse ones (Figures 2 and 3); (ii) an increased localization of Clec7a^+^ microglia around smaller, diffuse plaques, with a concomitant reduction in their association with larger, compact plaques (Figure 4); (iii) a marked protection from synapse loss in spite of similar amyloid burdens (Figure 5) and associated reduction in microglial‐mediated clearance of synaptic material (Figure 6). These observations, discussed in context below, emphasize a focus on plaque‐level heterogeneity and microglial responsiveness in control of amyloid‐associated tissue damage in the context of notable total amyloid burden. This highlights the potential of therapeutic targeting of PLCγ2 as an adjunct to other therapies, such as the targeting of amyloid.

Genetic study of AD identified the rare protective coding variant of *PLCG2* (P522R) associated with reduced risk of late‐onset AD (Sims et al. 2017). Given that *PLCG2* encodes an enzyme with potential as a drug target, it is important to define how this protective variant influences AD pathology. We and others have shown that PLCγ2^R522^ is hyperfunctional, enhancing intracellular Ca^2+^ signaling and modulating microglial endocytosis and phagocytosis (Maguire et al. 2021; Magno et al. 2019). To address the role of the AD‐protective PLCγ2 variant on the regulation of microglial behavior in vivo, we used a *Plcg2* ^*R522*^ knock‐in mouse model (Maguire et al. 2021). These were bred to the *App* ^*NL‐G‐F*^ AD‐like model to determine responses in the context of amyloid‐driven pathology (Saito et al. 2014).

*Plcg2* ^*R522*^ mice have increased microglial network coverage compared to *Plcg2* ^*P522*^ mice, in both dorsal hippocampus (CA1) and cortex. Microglia in *Plcg2* ^*R522*^ mice exhibited reduced ramifications and increased CD68 expression, indicative of a more reactive state (Figure 1). This aligns with a prior description of enhanced microglial activity in *Plcg2* ^*R522*^ expressing adult wildtype mice (Takalo et al. 2020). However, these authors did not report such alterations in microglial number or morphology, likely due to analysis of a small number of mice in contrast to our larger, sex‐considerate study. Given the role for PLCγ2 in AD, we investigated how *Plcg2* ^*R522*^ expressing microglia altered amyloid‐driven pathology (Sims et al. 2017; Bellenguez et al. 2022; van der Lee et al. 2019).

The *App* ^*NL‐G‐F*^ mice are characterized by progressive amyloid deposition from 2 months to cognitive decline by 6 months (Saito et al. 2014). Somewhat unexpectedly, the presence of the AD‐protective R522 variant did not reduce overall amyloid load, as total amyloid deposition (6E10 staining) was comparable between *Plcg2* ^*R522*^‐ and *Plcg2* ^*P522*^‐expressing mice. A more granular analysis of plaque composition revealed a phenotypic shift in amyloid plaques. ThioS and 4G8 antibody staining, which preferentially label compacted plaque cores, showed a generalized increase in *Plcg2* ^*R522*^ mice, while diffuse plaque pathology was reduced in the same mice when compared to *Plcg2* ^*P522*^ mice. These findings indicated that the impact of the R522 variant on microglia may manifest through promotion of plaque compaction.

We reanalyzed plaque composition using X34, which labels β‐sheet‐rich amyloid structures across both diffuse plaque‐associated amyloid and dense plaque cores, and confirmed that overall amyloid burden was similar between the *Plcg2* genotypes, consistent with 6E10‐staining. Fine stratification based on plaque size and compactness revealed a clear shift in plaque morphology, whereby *Plcg2* ^*R522*^‐expressing mice exhibited fewer small, diffuse plaques and an increased incidence of larger, highly compacted plaques. This points toward the protective variant influencing plaque maturation rather than overall amyloid accumulation. A shift toward increased plaque compaction is notable, as compact plaques are often considered more structurally stable and less prone to dispersion than diffuse plaques, which are more associated with higher toxicity. These findings align with previous reports suggesting that plaque morphology, rather than total amyloid burden, may be a key factor in neurotoxicity and disease progression and suggest that this could be an important role for plaque‐associated PLCγ2 expressing microglia (Casali et al. 2020; Shi et al. 2017; Yuan et al. 2016).

We next examined the microglial response associated with different plaque types. In *Plcg2* ^*R522*^‐expressing mice, Iba1^+^ microglia exhibited a stronger peri‐plaque response around smaller, lower‐compactness plaques, while microglial engagement was reduced in compacted plaques. This suggests a heightened response to diffuse plaques but reduced microgliosis around highly compacted plaques. Given that the reactive microglia state extends beyond changes in density and spatial distribution, we analyzed the expression of “homeostatic” and “DAM” markers, which distinguish functionally distinct microglial states. Tmem119, a homeostatic marker that is generally downregulated within plaque niches, was found to be further reduced in compact plaques in *Plcg2* ^*R522*^‐expressing mice. A similar pattern was observed for total Tmem119 expression, aligning with our Iba1^+^ microgliosis data, showing that microglia were more engaged with diffuse plaques but less so with highly compacted plaques. In contrast, Clec7a is a DAM marker exclusively expressed at plaques. Microglia in *Plcg2* ^*R522*^‐expressing mice exhibited higher total Clec7a expression around smaller, diffuse plaques, while significantly reduced Clec7a expression was observed in the largest, most compacted plaques. These findings reinforce the finding that *Plcg2* ^*R522*^‐expressing microglia are more reactive to diffuse plaques but exhibit a reduced response to compacted plaques, indicating a potential shift in microglial function and plaque interaction dynamics. We also re‐examined the role of PLCγ2 in regulation of the inflammatory cytokine response. Results from in vivo mouse models have not been consistent. Here, we took the opportunity to use the more disease relevant human iPSC‐derived microglia. There were clear differences between the *PLCG2* variants, but notably also, marked differences depending on the stimulus (Aβ versus LPS). While we observed an in vivo increase in TNF in the *App* ^*NL‐G‐F*^ mice expressing the *Plcg2* ^*R522*^ variant and also an enhanced TNF response to Aβ, by the human cells with the same variant, the LPS response was evidently different, and hence different to results previously reported with mouse cells in vitro. In vivo context is essential for relevant microglial responses, but all the mouse models are confounded by different disease progression and hence level and nature of challenge.

Importantly, and in spite of this consistent abundance of amyloid, *Plcg2* ^*R522*^‐expressing mice were significantly protected against amyloid‐induced synapse loss, as evidenced by preserved synaptic puncta and dendritic spine density and reduced evidence of phagocytosis of synaptic material in vivo. These observations underscore a potential neuroprotective role of microglia modulated by PLCγ2. These initial observations of a statistically significant protection from synapse loss of the R522 variant of PLCγ2 were replicated in the cortex and when the two brain regions were divided based on plaque proximity.

While we have previously demonstrated that PLCγ2‐R522 alters microglial endocytic and phagocytic activity, including modest enhancement of β‐amyloid uptake in vitro (Maguire et al. 2021), the largely unchanged total amyloid burden observed here suggests that altered amyloid clearance is unlikely to be the dominant mechanism of protection in the *App* ^*NL‐G‐F*^ model. Instead, our findings support a model in which PLCγ2 reshapes plaque architecture and plaque‐associated microglial responses, with downstream preservation of synaptic integrity.

Our findings contrast with the results of others studying the P522R variant in the 5xFAD AD model. The 5xFAD model, like many other models, relies on APP overexpression and demonstrates an elevated Aβ42:40 ratio (~2–3:1) (Oakley et al. 2006). The *App* ^*NL‐G‐F*^ knock‐in model, however, has physiological *App* expression levels, avoiding the overexpression artifacts found in other transgenic models, but is strongly biased toward very high Aβ42:40 ratios (~10–100:1) because of the familial mutations, which developed with age alongside increasing amyloid pathology (Saito et al. 2014). Such limitations are present with all mouse models of AD, balancing artificial expression with familial mutations that drive pathological processes. In the 5xFAD context, a reduction in plaque load was attributed to the PLCγ2 AD‐protective variant (Tsai et al. 2023). The impact of PLCγ2 variants on the amyloid burden in the 5xFAD model underscores the potential importance of PLCγ2 activity in regulating amyloid processing but limited the authors' ability to interpret the role of the variants in downstream pathological sequelae, such as the impact on neuronal function. Nonetheless, in the 5xFAD background, in the context of reduced amyloid burden, the PLCγ2^R522^ variant was associated with a clear protection of synaptic function and working memory, the latter measured by Y‐maze alternation. During submission of this manuscript, another study (Takalo et al. 2025), using the APP/PS1 model which develops an elevated Aβ42:40 (~5:1) (Radde et al. 2006), proposed that the protective variant reduced the number of plaques and overall plaque load, again confounding downstream studies; however, the remaining plaques which were not obviously altered (as scored by gross plaque X34 and 6E10 staining loads). The authors associated the effects of the protective R522 variant of PLCγ2 with changes in microglial activity (Takalo et al. 2025). In this case, in the APP/PS1 model, no obvious impact of the R522 variant of PLCγ2 was observed in learning and memory tests, but an alteration in anxiety was suggested, which was exacerbated by disease but also, interestingly, indicated without. This proposed cognitive change in PLCγ2‐R522 expressing mice independently of a disease model is interesting in the light of our finding of a subtle alteration in the synaptic network of these mice. While we have not conducted behavioral studies here, our synaptic studies allow fine study of the spatial impact of tissue damage on the underlying cognitive networks. It would be prudent to consider any pre‐existing conditions with these mice in future studies. With regard to amyloid burden, in our studies, at the gross level, staining of X34 and 6E10 detected no clear difference between the genotypes; however, we identified the aforementioned differences in plaque morphology associated with *Plcg2* genotype. Additionally, as detailed above, these plaque differences were intimately related to their association with microglia.

While our primary focus was on the role of PLCγ2 on disease pathology, the experimental design afforded an examination of the influence of sex. Broadly summarized, we observed increases in cortex plaque compaction associated with females and increased diffuse plaque presence in the hippocampus of males (Figures 2 and S2). Interestingly, a protective effect of the R522 PLCγ2 variant distal from the plaques in the cortex was significantly demonstrated only in the male mice, which experience lower plaque compaction than females in this region. While our experiments were appropriately sized, these specific interactions between sex and genotype could reflect false discovery. Sex differences in animal models are often understudied and these observations indicate that a more detailed study of pathology development in this model is merited. Additionally, an in vitro study with iPSC has suggested that there may be a gene dosage effect of the variant (Solomon et al. 2022), although this was not the focus of our study.

Collectively, our studies indicate an uncoupling of total amyloid burden and synaptic loss mediated by PLCγ2. Specifically, this is characterized by a largely unaltered total amyloid burden that is associated with altered microglial plaque responses and skews in plaque morphology. This novel insight suggests that modulation of amyloid‐associated microglial activation can directly regulate synaptic clearance, perhaps via alteration of plaque constitution. This consolidates the case for therapeutic targeting of PLCγ2 for neuroprotection and cognitive resilience in AD. Consistent with this, and demonstrating the relevance of our observations to human disease and aging, the R522 variant of PLCγ2 is in humans associated with longevity and protection of cognitive function in old age and protects against AD, as well as frontotemporal dementia and dementia with Lewy bodies (Sims et al. 2017; van der Lee et al. 2019; Strickland et al. 2020). Furthermore, cognitive decline itself is poorly correlated with changes in amyloid burden and significant numbers of aged cognitively normal individuals exhibit some level of amyloid deposition, suggesting that other mechanisms drive symptom development (Bourgeat et al. 2010; Aizenstein et al. 2008; Sturchio et al. 2022). The first therapies for AD targeting the removal of amyloid are now being approved with modest positive outcomes, although associated with several side effects (Sims et al. 2023; Mintun et al. 2021; Van Dyck et al. 2023).

In summary, the R522 AD protective variant of PLCγ2 promotes a marked neuroprotection characterized by retention of essentially normal synaptic densities in *App* ^*NL‐G‐F*^ mice. This synaptic protection was associated with a notable reduction in phagocytic clearance of synaptic materials, occurred in the context of similar plaque loads and was associated with changes in plaque structure and microglial involvement. While therapeutic manipulation of PLCγ2 may operate at the level of plaque formation and/or clearance (as also supported in other models) (Tsai et al. 2023; Takalo et al. 2025), our findings indicate that PLCγ2 can mediate dramatic protection of synapses from amyloid‐driven synapse loss. Therapeutically PLCγ2‐targeting would provide an alternative strategy that could be employed independently of, or as an adjunct to, current amyloid‐directed approaches and may have broad applicability in neuroinflammatory conditions beyond the scope of AD.

## Author Contributions

**Ryan J. Bevan:** conceptualization (supporting), data curation (lead), formal analysis (lead), investigation (lead), methodology (lead), project administration (supporting), validation (equal), visualization (equal), writing – original draft (equal), writing – review and editing (equal). **Emily Maguire:** data curation (supporting), formal analysis (supporting), investigation (supporting), methodology (supporting), validation (equal), writing – review and editing (equal). **Eilish Mackinnon:** data curation (supporting), formal analysis (supporting), investigation (supporting), methodology (supporting), validation (equal), visualization (supporting), writing – review and editing (supporting). **Thomas Phillips:** investigation (supporting), methodology (supporting), writing – review and editing (supporting). **Elisa Salis:** data curation (supporting), formal analysis (supporting), investigation (supporting), methodology (supporting), validation (equal), visualization (supporting), writing – review and editing (supporting). **Elena Simonazzi:** investigation (supporting), methodology (supporting), writing – review and editing (supporting). **Marieta Vassileva:** investigation (supporting), methodology (supporting), writing – review and editing (supporting). **Nicholas D. Allen:** methodology (supporting), resources (supporting), supervision (supporting), writing – review and editing (supporting). **Julie Williams:** resources (supporting), supervision (supporting), writing – review and editing (supporting). **Philip R. Taylor:** conceptualization (lead), formal analysis (supporting), funding acquisition (lead), project administration (lead), resources (lead), supervision (lead), validation (equal), visualization (equal), writing – original draft (equal), writing – review and editing (equal).

## Funding

This work was supported by the UK Dementia Research Institute (DRI‐3203) and Moondance Foundation.

## Ethics Statement

All experiments were approved by the Animal Welfare and Ethical Review Body—subgroup of the Biological Standards Committee—and conducted in accordance with UK Home Office Guidelines and Animal [Scientific Procedures] Act 1986, which encompasses EU Directive 2010/63/EU on the protection of animals used for scientific purposes.

## Conflicts of Interest

The authors declare no conflicts of interest.

## Supporting information


**Figure S1:** Plcg2^R522^ variant alters amyloid burden in the hippocampus of App^NL‐G‐F^ mice.
**Figure S2:** Amyloid plaque burden characterized in Plcg2^R522^ variant expressing and control mice with ThioS.
**Figure S3:** Plcg2^R522^ variant effects on inflammatory responses.
**Figure S4:** Plcg2^R522^ expression protects hippocampal and cortical synapses in App^NL‐G‐F^ mice.
**Figure S5:** Examples of DiOlistic labelling of hippocampal dendritic spines.

**Video S1:** Imaris rendered animation of peri‐plaque engulfment of synaptic puncta in App^NL‐G‐F^ mice.

## Data Availability

The data that support the findings of this study are available from the corresponding author upon reasonable request.
