# Supplementary material for: Alzheimer's Protection by PLCγ2 Compacts Plaques, Redistributes Microglia, and Protects Synapses in App NL ‐G‐F Mice
Source: Glia. 2026 Jun 25;74(8):e70192. doi: 10.1002/glia.70192 (PMC13305147; doi:10.1002/glia.70192)
Supplement: Supplementary file 1 — Figure S1: Plcg2R522 variant alters amyloid burden in the hippocampus of AppNL‐G‐F mice. Figure S2: Amyloid plaque burden characterized in Plcg2R522 variant expressing and control mice with ThioS. Figure S3: Plcg2R522 variant effects on inflammatory responses. Figure S4: Plcg2R522 expression protects hippocampal and cortical synapses in AppNL‐G‐F mice. Figure S5: Examples of DiOlistic labelling of hippocampal dendritic spines. [file GLIA-74-0-s001.pdf]

SUPPLEMENTARY DATA

Alzheimer′s protection by PLCγ2 compacts plaques, redistributes microglia, and protects synapses in *App*$^{NL-G-F}$ mice

Ryan J. Bevan[1,2], Emily Maguire[1], Eilish Mackinnon[1,2], Elisa Salis[1], Thomas Phillips[1,2], Elena Simonazzi[1,2], Marieta Vassileva[1,2], Nicholas D. Allen[3], Julie Williams[1] and Philip R. Taylor[1,2].

Author affiliations:

[1] UK Dementia Research Institute at Cardiff University, Cardiff University, Cardiff, CF24 4HQ, UK.

[2] Systems Immunity Research Institute, Heath Park, Cardiff University, Cardiff, CF14 4XW, UK.

[3] School of Biosciences, Cardiff University, Cardiff, CF10 3AX, UK.

Correspondence to:

Professor Philip R. Taylor

UK Dementia Research Institute at Cardiff University,

Hadyn Ellis Building, Maindy Road, Cardiff University, Cardiff, CF24 4HQ, UK;

E-mail: TaylorPR@Cardiff.ac.uk

Supplementary Methods

Immunofluorescence and Image Acquisition Settings

In both the hippocampus CA1 region and overlaying cortices, Iba1$^+$ cell bodies, 6E10/4G8 plaques and ThioS$^+$ plaque coverage were imaged from three non-overlapping fields within the same coronal section per mouse using the 10x objective, scan speed of 600Hz, line average of 3 covering a field of view size of 1,500 µm x 1,500 µm (voxel size of 0.379 µm) through a depth of 60 µm with intervals of 5 µm. Images were compressed to a 2D maximum projection for analysis.

Images of amyloid plaque labelling and associated colabelling from the hippocampus CA1 region were captured using a 20x objective from three non-overlapping fields within the same coronal section per mouse, scan speed of 600Hz, and line average of 2 covering a field of view size of 775 µm x 775 µm (voxel size of 0.379 µm) through a depth of 10 µm with intervals of 0.695 µm and images compressed to a 2D maximum projection for analysis.

For microglial morphology, three images per mouse from the hippocampus CA1 regions were captured using a 40x objective to generate an analysis for 10 isolated single microglia per mouse, scan speed of 600Hz, and line average of 3 covering a field of view size of 388 µm x 388 µm (voxel size of 0.189 µm) through a depth of 40 µm with images analysed as 3D stacks.

For punctate staining in the hippocampus CA1 region, CD68 and synaptic puncta up to 10 images per mouse were captured using the 63x objective, scan speed 600Hz, line average of 3 covering a field of view size of 246 µm x 246 µm (voxel size of 0.06 µm) through a depth of 5 µm.

For synaptic puncta staining alone, only a middle depth of 1 µm from the initial 5 µm was analysed to generate six 30 µm x 30 µm regions for data analysis, for CD68 the entire 5 µm stack was analysed from 10 microglia, for plaques/CD68 including containing with synaptic puncta, the entire 5 µm stack was analysed from 5 plaque images. Images were pseudo-coloured for visualisation of immunostaining.

For DiOlistics, Leica SP8 Lightning confocal microscope was configured under the resonant scanner with PMT photodetector set with a gain of 400 and fixed laser powers. Spines images, up to 10 per mouse, were captured using 63x objective, scan speed of 8000Hz, line average of 2 covering a field of view size of 147.72 µm x 147.72 µm (voxel size of 0.144 µm) through a varying z depth typically spanning 20-50 µm dependent on the labelling.

Immunofluorescence and Image Analysis

All images were analysed using Imaris (v10.0, Bitplane) and batch-processed by combining them into a "time-series" for each experimental marker. This preprocessing step included ROI selection, background/threshold adjustments, and Gaussian/median filtering, followed by analysis using the Imaris Batch function.

For 10x and 20x objective images, regions of interest (ROIs) within the hippocampal CA1 stratum radiatum and, where indicated, the full cortical thickness directly overlying the hippocampal CA1 region, were manually delineated using the Surface tool. ROIs encompassed the entire tissue region of interest within each image to control for tissue size variations in data interpretation. For example, when assessing hippocampal CA1-specific changes, analysis was restricted to the CA1 ROI, with adjacent tissue regions excluded.

For 40x objective 10 microglia morphology images and 63x objective CD68 puncta in microglia, single microglia territory fields were extracted from the original images based on presence of DAPI nuclei and representing single microglia. For 63x objective synaptic puncta staining, 6 smaller ROI fields 30 µm x 30 µm (excluding cell nuclei and greater than 30 µm from plaques where relevant) were positioned and extracted from the original images. For 63x peri-plaque analysis, circle ROI territory fields (30 µm radius, number specified in the figure legend) were extracted over AmyloGlo plaque cores and extracted from original images.

In all analysis pipelines the fluorescence intensities across the extracted regions were normalised across the batch 'time-series' using the Imaris XTension 'Normalise Time Points', applied with a Gaussian or median filter and background subtracted using the 'Linear Stretch' function. For 10x objective images, Iba1^{+} cell bodies were quantified using the Spot function and 6E10/4G8^{+} and ThioS^{+} plaques were identified using the Surface function. For 6E10/4G8^{+} analysis, 6E10^{+} plaques were identified and masked onto the 4G8^{+} channel to classify plaque core phenotypes. For 20x objective images of X34 plaques, co stained with Iba1/Tmem119, Iba1/Clec7a, Tmem119/Clec7a, X34 plaques and Iba1 labelling were surface rendered with the Iba1 signal subclassified as peri-plaque if less than 5 µm from plaque borders. Intensity measures of Tmem119^{+} and Clec7a^{+} were obtained from the Iba1^{+} mask. For 40x objective images of microglia morphology, the Surface function was used to 'isolate' and quantify single microglia based on the largest surface within the image and the presence of single DAPI nuclei. Single microglia surfaces, 10 microglia per mouse, were then extracted and morphologically assessed using the Filament Tracer function, with the Imaris inbuilt AI segment features based on the seed point of 0.2 µm and AI features from the images by assigning segments that were

correctly or incorrectly mapping the microglia processes for individual microglia. Traced microglia per mouse generated mean morphometric scores for each mouse representing the sholl analysis area under the curve (AUC) and number of terminal points.

Antibody detection of amyloid plaque analysis for global measures from 10x images were subtyped based on their morphological phenotype based on the presence/absence of 6E10^{+} and 4G8^{+} labelling. X34 plaque labelling from 20x images were subtyped based on their morphological phenotype first based on their respective diameters (Imaris- Bounding box C) into 4 groups, 5-10 µm, 10-20 µm, 20-40 µm, and greater than 40 µm. Within each of these size groups, plaques were classified based on their relative compactness measured by the mean intensity of the X34 signal, grade 1 corresponding to diffuse/very limited presence of core, grade 2 intermediate compactness plaques containing a defined core with surrounding diffuse amyloid, and grade 3 plaques that were primarily core with very limited surrounding diffuse amyloid. This approach enabled a thorough investigation into the plaque heterogeneity peri-plaque resolution in the models.

For 63x objective images of microglia with CD68 puncta, images were analysed using the surface function to isolate the microglia channel image and masked onto the CD68 channel, with the CD68 puncta quantified with the surface function. For 63x objective images for synaptic puncta, punctate staining was analysed using the Surface function. For 63x objective images for peri-plaque analysis, isolated plaques falling within the 10-20 µm diameter bracket with presence of cores (based on AmyloGlo labelling) from circle ROI territory fields (30 µm radius) were surface rendered to isolate the plaque and microglia immunoreactivity, with the microglia surface render masked onto the CD68 channel and, where relevant, the CD68 channel masked onto the synaptic puncta channel for assessing synaptic engulfment. Synaptic puncta staining was surface rendered for the original puncta staining channel and the masked engulfed synaptic puncta that colocalise with CD68 within microglia. An animation of the engulfed synaptic puncta peri-plaque in $App^{NL\text{-}G\text{-}F}$ mice is demonstrated in **Supp Video 1**.

For dendritic spine analysis, Imaris (v9.9, Bitplane), the dendrite base shaft was traced using default thresholding followed by spine tracings using fixed parameters of spine head seed point size of 0.45 µm and manually checked to ensure correct tracing. Spines were automatically subclassified (stubby, mushroom, thin) using SpineClassifier MATLAB extension, distinguishing based on spine length and head size with the following fixed parameters: Stubby = length(spine) < 0.8 [short protrusions without presence of spine neck], Mushroom = length(spine) < 3 and max_width(head) > mean_width(neck) [spine protrusions with defined

and obvious spine head], Filopodia = length(spine) > 3 [this spine subtype was removed from analysis due to infrequent occurrence at this age point], and Thin = true [all remaining spines].

Synaptic puncta co-labelling with X34-positive plaques and quantification

Free-floating brain sections prepared and stained as above and were processed for additional immunofluorescence analysis of synaptic integrity. Sections were stained with X34 to detect amyloid plaque material, which in *App*$^{NL\text{-}G\text{-}F}$ mice robustly labels both diffuse and compacted plaques across a broad intensity range. X34-labelled plaques were co-stained with Bassoon and PSD95 to assess presynaptic and postsynaptic puncta colocalisation within the CA1 stratum radiatum of the hippocampus and the mid-layers (IV-VI) of the overlying cortex. For each animal, three fields were imaged per region using a Leica SP8 Lightning confocal microscope with a 63x objective (1 µm z-depth, voxel size 0.06 µm), with identical laser and detector settings applied across all groups.

All images were analysed in Imaris (v10.0, Bitplane) and batch-processed as described above, with adaptations to allow for large-scale puncta analysis. In brief, images were combined into a pseudo-time series for batch processing, including ROI selection of the entire CA1 or cortical region, background subtraction, thresholding, and Gaussian filtering. X34 plaques were surface rendered, and adjacent plaques within 2 µm were merged using the distance transformation function to ensure continuous plaque territories rather than fragmented surfaces. This transformation also generated a distance-intensity channel, in which intensity values reflect proximity to plaque borders (e.g., intensity = 0 at plaques, 5 at 5 µm from the border, 30 at 30 µm, etc.).

Following this step, images were separated back into individual files and batch processed using the Spot function for puncta detection to generate the following readouts: (1) X34 plaque territories (from the distance transformation channel), (2) Bassoon-positive puncta within 5 µm of X34 plaques, (3) PSD95-positive puncta within 5 µm of X34 plaques, (4) Bassoon-PSD95 colocalised puncta within 0.3 µm of each other in the peri-plaque region, (5) Bassoon-positive puncta >30 µm from plaques, (6) PSD95-positive puncta >30 µm from plaques, and (7) Bassoon–PSD95 colocalised puncta >30 µm from plaques. All puncta data were reported as densities per mm^2 of tissue. For distal measures, puncta >30 µm from plaques were quantified across the defined tissue ROI. For peri-plaque measures, puncta were spatially mapped to their corresponding X34 plaques, and analyses were restricted to plaques within a defined diameter range, with the largest consistent subset of plaques across images selected for comparison. Imaging and analysis were performed in a single batch with uniform processing parameters.

All derived data were analysed using linear mixed-effects models, with *Plcg2* variant (P522 vs. R522) and sex included as fixed factors, and replicate within mouse as random effects.

Total RNA extraction, cDNA synthesis and RT-QPCR

Snap-frozen cortical tissue (~50 mg) was homogenised in QIAzol lysis reagent (Qiagen, 79306) using a motorised pestle, and total RNA was extracted with the miRNeasy Micro Kit (Qiagen, 217084) according to the manufacturer′s instructions. Genomic DNA was removed with the Turbo DNA-free Kit (Invitrogen, AM1907) prior to cDNA synthesis using the High-Capacity cDNA Reverse Transcription Kit (Invitrogen, 4374967). No reverse transcription (RT) controls were prepared for each sample.

Gene expression was quantified by RT-qPCR using FAST SYBR Green Master Mix (Thermo Fisher, 4385612), with three technical replicates per reaction, and normalised to GAPDH using the ΔΔCt method. The following primer sequences were used: *Clec7a*: 5′-CCAGCTAGGTGCTCATCTACTG-3′ and 5′-CTTCACTCTGATTGCGGG-3′; *Tnf*:5′-GTCCCCAAAGGGATGAGAAGTT-3′ and 5′-GTTTGCTACGACGTGGGCTACA-3′; *Il6*: 5-AGATAAGCTGGAGTCACAGAAGGAG-3′ and 5′-CGCACTAGGTTTGCCGAGTA-3′; *Gapdh*: 5′-TGGCAAAGTGGAGATTGTTGCC-3′ and 5′-AAGATGGTGATGGGCTTCCCG-3′.

Generation of iPSC-derived microglia

We have previously described the generation, culture and differentiation of PLCG2 variant human induced pluripotent stem cells (hiPSC)[1]. iPSCs were differentiated into microglia using a protocol according to Haenseler et al.[2]. Briefly, iPSCs were treated with 10 µM Y-27632 for 1 h prior to dissociation with Accutase and seeded into ultra-low attachment 96-well plates at 1×10^4 cells per well in Essential 8 Flex medium (Thermo Fisher) supplemented with 10 µM Y-27632 (Tocris), 50 ng/mL BMP4, 50 ng/mL VEGF121 and 50 ng/mL SCF (Peprotech). Embryoid bodies (EBs) were maintained for 4 days, including a 50% medium change on day 2.

On day 4, EBs were transferred to X-VIVO15 medium (Lonza) supplemented with 1% Penicillin/Streptomycin, 1% GlutaMAX, 50 nM β-mercaptoethanol, (Thermo Fisher) 50 ng/mL M-CSF and 50 ng/mL IL-3 (Peprotech). Two-thirds of the medium was replaced every 5–7 days. After approximately 25 days, non-adherent microglial precursor cells (MPCs) were

harvested weekly and differentiated into microglia by culture in astrocyte-conditioned medium (ACM) for 12–14 days, with a complete medium change after 7 days.

ACM was generated from iPSC-derived astrocytes differentiated as described below. Briefly, mature astrocytes were cultured in Advanced DMEM/F-12 (Thermo Fisher) for 48 h, after which conditioned medium was collected, pooled, filter-sterilised and stored at −80°C. ACM batches were analysed for CCL2 content by ELISA (Bio-Techne) and normalised to a final CCL2 concentration of 1 ng/mL prior to use[1].

Generation of iPSC-derived astrocytes and astrocyte-conditioned media

Human iPSCs were differentiated into astrocytes using a protocol adapted from Serio et al.[3]. Briefly, iPSCs were first differentiated into neural progenitor cells (NPCs) according to Telezhkin et al.[4] using dual SMAD/WNT inhibition (SB431542, 10 µM; LDN193189, 200 nM, IWR-1-endo, 1.5 µM (Tocris)) in Advanced DMEM/F-12 supplemented with NeuroBrew-21 without retinoic acid (Miltenyi). Following neural induction, NPCs were maintained until day 16 in neural maintenance medium.

NPCs were subsequently expanded for three passages in astrocyte progenitor cell (APC) medium comprising Advanced DMEM/F-12 supplemented with NeuroBrew-21, EGF (20 ng/mL) and LIF (20 ng/mL) (Peprotech). APCs were enriched by fluorescence-activated cell sorting using a CD44 antibody (Miltenyi Biotec), with gates established using the corresponding isotype control. CD44-positive APCs were expanded and cryopreserved in CryoStor CS10 (StemCell Technologies) as required.

For terminal differentiation, APCs were cultured on Matrigel-coated plates (Corning) in a 1:1 mixture of Neurobasal-A and Advanced DMEM/F-12 supplemented with CNTF (10 ng/mL, Peprotech) for 14 days, with a medium change after 7 days. For ACM production, mature astrocytes were washed with PBS and then cultured in Advanced DMEM/F-12 for 48 h. Conditioned medium was collected, centrifuged to remove cellular debris, filtered through a 0.22 µm membrane, aliquoted and stored at −80°C until use.

Stimulation of iPSC-derived microglia-like cells

For stimulation of the cells, with Aβ, lyophilized $A\beta_{1\text{-}42}$ (Bachem, #4061966.1) was dissolved in 500 µl of Hexafluoroisopropanol (HFIP), vortexed thoroughly for 30 seconds before being dried under a stream of nitrogen gas. The dried pellet was resuspended in 500 µl of HFIP and sonicated in a bath sonicator for 5 minutes before being dried again with a SpeedVac Vacuum

centrifuge. Finally the Aβ pellet was resuspended in PBS, aliquoted and stored at -80 °C until use. LPS was obtained from Sigma-Aldrich (#L6529).

For the LPS and Aβ challenge, macrophage precursors were seeded at 10^4 cells per well in fibronectin coated 96-well plates and cultured for 14 days. Cells were stimulated with either with 100 ng/ml of LPS or 5 µM of Aβ for the indicated times and then supernatant was harvested and frozen for future analysis. Levels of IL-6, IL-12p40, IL-10, TNF and G-CSF were determined using LEGENDplex Custom Human kit (Biolegend), following manufacturer's instructions. Data were collected using a LSR Fortessa™ (BD Biosciences) and analysed using LEGENDplex TM v8.0 (Biolegend). Analyses include three independent experiments and each independent experiment was conducted with 3 independent clones for each genotype. Data were normalized to the wild type (P522) response to facilitate comparison of independent experiments. Data were analysed by two-way ANOVA and indicated genotype effects were analysed with Holm-Sidak's post-tests.

Supplementary References.

1. Maguire et al., 2021. "PIP2 depletion and altered endocytosis caused by expression of Alzheimer's disease-protective variant PLCγ2 R522". *EMBOJ* **40**(17):e105603. doi: 10.15252/embj.2020105603.

2. Haenseler et al., 2017. "A Highly Efficient Human Pluripotent Stem Cell Microglia Model Displays a Neuronal-Co-culture-Specific Expression Profile and Inflammatory Response". Stem Cell Reports **8**:1727-1742.

3. Serio et al., 2013. "Astrocyte pathology and the absence of non-cell autonomy in an induced pluripotent stem cell model of TDP-43 proteinopathy". *Proc Natl Acad Sci U S A* **110**, 4697-702.

4. Telezhkin et al. 2016. "Forced cell cycle exit and modulation of GABAA, CREB, and GSK3β signaling promote functional maturation of induced pluripotent stem cell-derived neurons". Am J Physiol Cell Physiol. **310**:C520-41. doi: 10.1152/ajpcell.00166.2015.

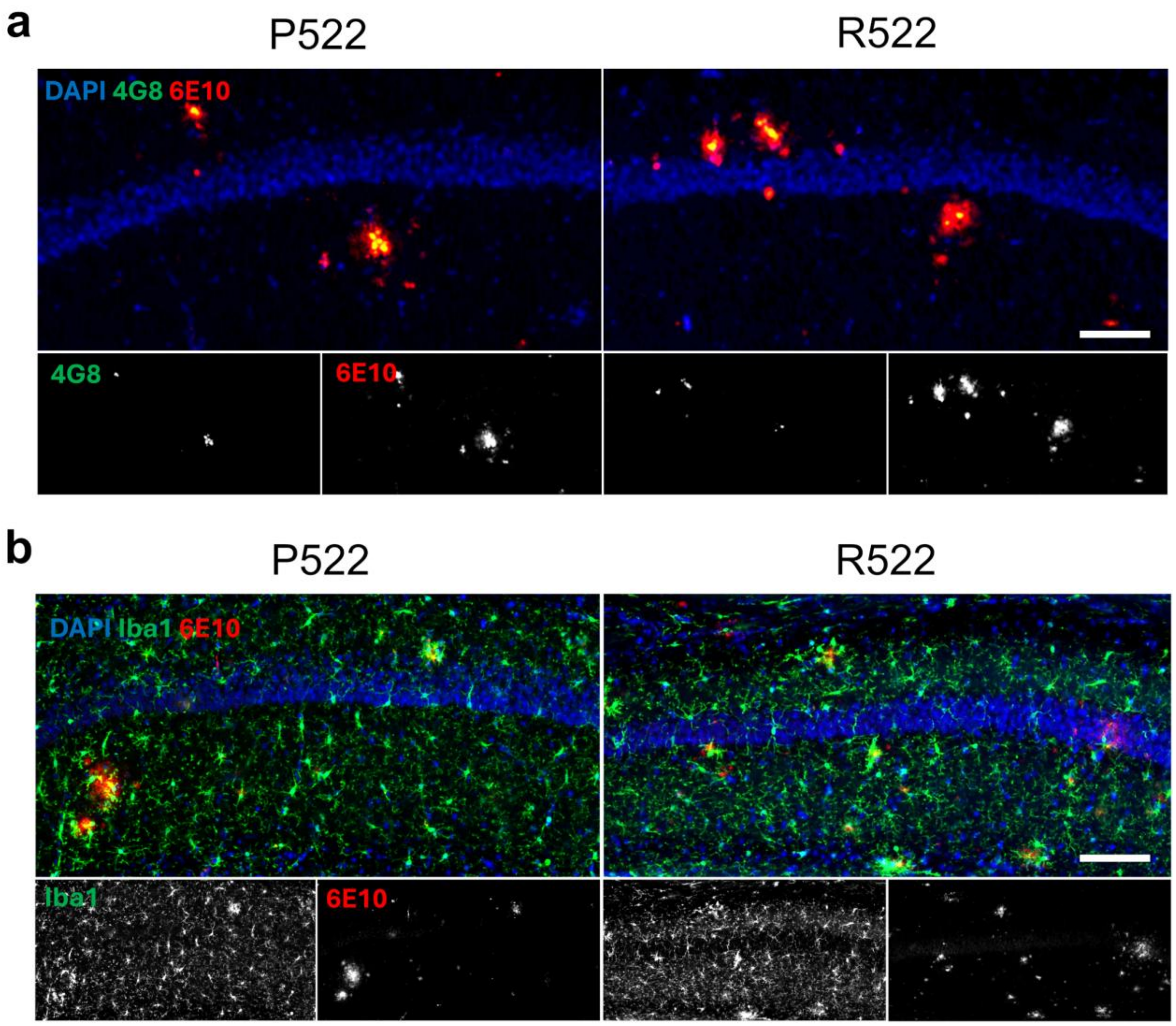

Supp Fig. 1. Plcg2^{R522} variant alters amyloid burden in the hippocampus of App$^{NL\text{-}G\text{-}F}$ mice. a *Representative images of 6E10-labelled plaques (red) and associated 4G8+ plaque cores (green) from the hippocampus (CA1) App$^{NL\text{-}G\text{-}F}$ mice expressing either Plcg2^{P522} or Plcg2^{R522} variant. Scale bar: 100 µm.* ***b*** *Representative images of 6E10-labelled plaques (red) co-stained with Iba1+ microglia (green) from the hippocampus (CA1) App$^{NL\text{-}G\text{-}F}$ mice expressing either Plcg2^{P522} or Plcg2^{R522} variant. Scale bar 100 µm.*

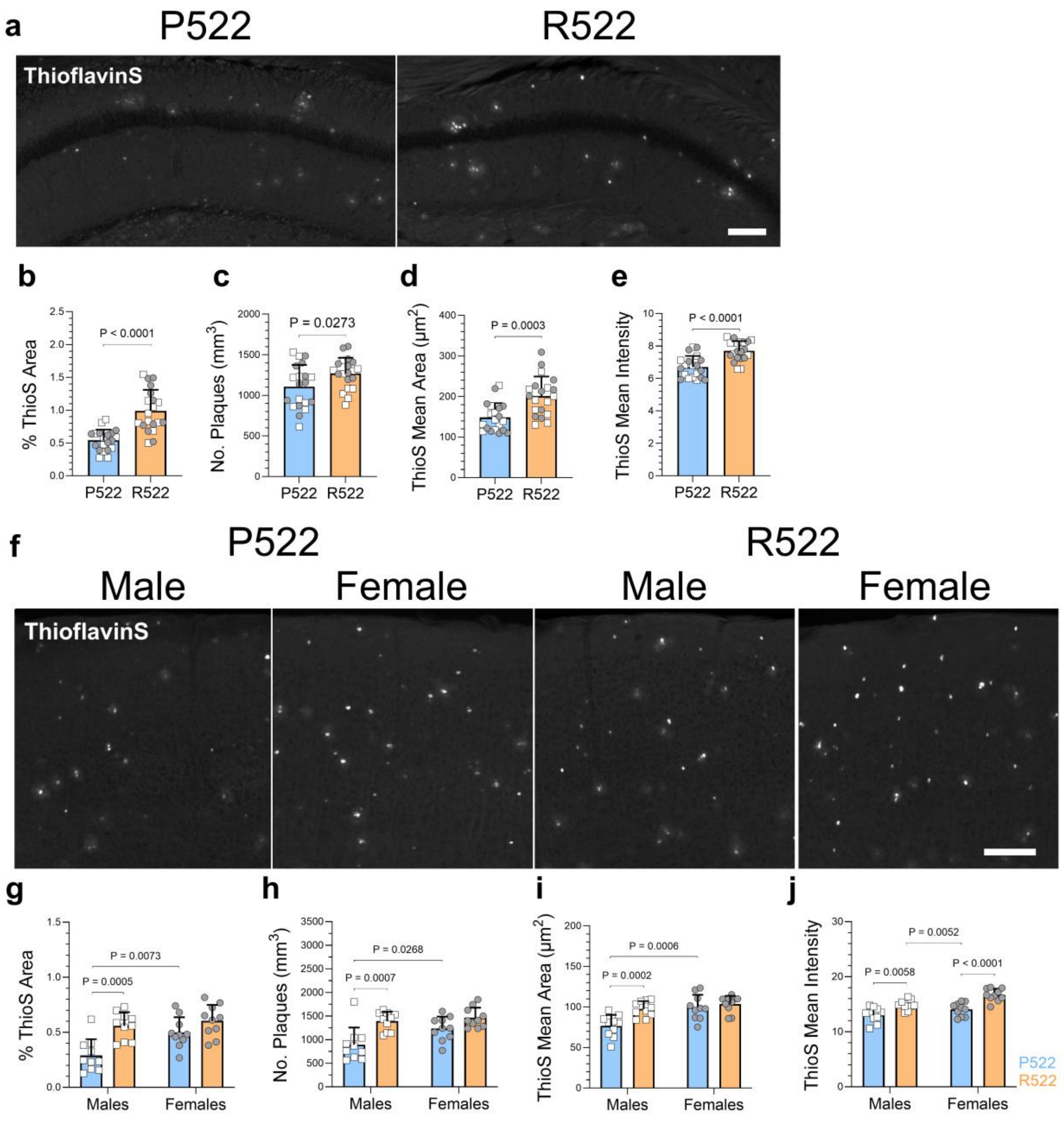

Supp Fig. 2. Amyloid plaque burden characterised in Plcg2^{R522} variant expressing and control mice with ThioS. a *Representative images of Thioflavin-S (ThioS) plaque deposition (white) from the hippocampus (CA1) of App$^{NL\text{-}G\text{-}F}$ mice expressing either Plcg2^{P522} or Plcg2^{R522} variant. Scale bar 100 µm.* ***b-e*** *Hippocampal plaque parameters: ThioS plaque area coverage, Number of ThioS plaques, Individual ThioS plaque core size and ThioS plaque intensity.* ***f*** *Representative images of Thioflavin-S (ThioS) plaque deposition (white) from the cortex of App$^{NL\text{-}G\text{-}F}$ mice expressing either Plcg2^{P522} or Plcg2^{R522} variant. Scale bar 100 µm.* ***g-j*** *Cortex plaque parameters: ThioS plaque area coverage, Number of ThioS plaques, Individual ThioS plaque core size and ThioS plaque intensity. All data points represent individual mice; N = 10 males (squares) and 10 females (circles) from both genotypes. Data represented as the average of 3 entire CA1 stratum radiatum hippocampal or cortex fields viewable within 2.40 mm^2 field of view (10x objective). Data were analysed by Two-way ANOVA considering*

genotype and sex with post hoc multiple comparisons test (Bonferrnoni); sex differences were detected for the cortex datasets. ***b-e*** *P values represent the effect of genotype (Plcg2 R522).* ***b*** *Interaction P = 0.6898, Sex P = 0.4900, Genotype P < 0.0001.* ***c*** *Interaction P = 0.2533, Sex P = 0.0920, Genotype P = 0.0273.* ***d*** *Interaction P = 0.2497, Sex P = 0.2895, Genotype P = 0.0003.* ***e*** *Interaction P = 0.4922, Sex P = 0.8689, Genotype P < 0.0001.* ***g*** *Interaction P = 0.0724, Sex P = 0.0062, Genotype P = 0.0001.* ***h*** *Interaction P = 0.1178, Sex P = 0.0186, Genotype P < 0.0001.* ***i*** *Interaction P = 0.0353, Sex P = 0.0007, Genotype P = 0.0032.* ***j*** *Interaction P = 0.2649, Sex P = 0.0005, Genotype P < 0.0001.*

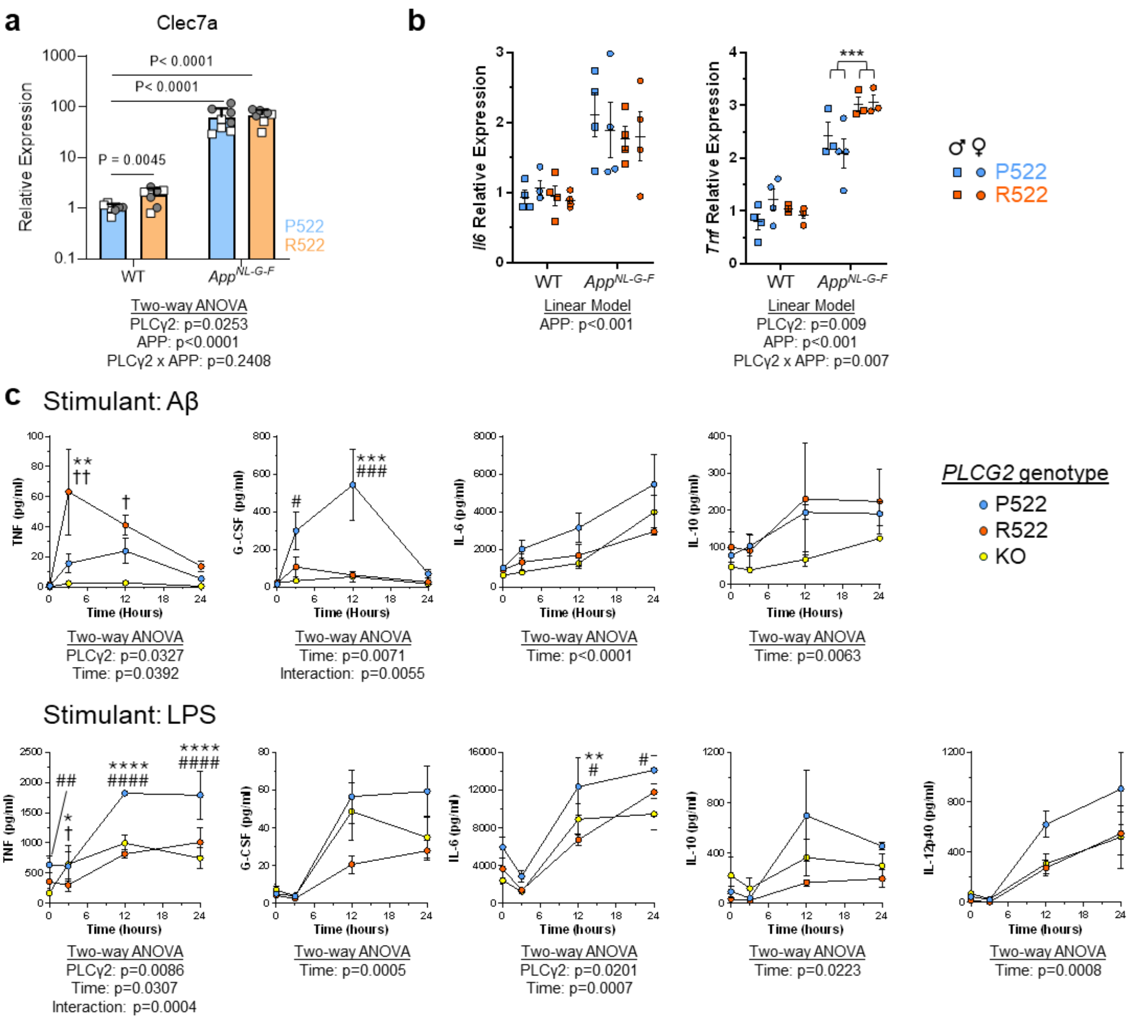

Post-test	P<0.05	P<0.01	P<0.001	P<0.0001
P522 vs R522	*	**	***	****
P522 vs KO	#	##	###	####
R522 vs KO	†	††	†††	††††

Supp Fig. 3. Plcg2^{R522} variant effects on inflammatory responses. a-b *Relative marker expression was measured by RT-qPCR in the cortex of wildtype (WT) and App$^{NL\text{-}G\text{-}F}$ mice expressing either the Plcg2^{P522} or Plcg2^{R522} variant. All data points represent individual mice from males (squares) and females (circles) across all genotypes.* ***a*** *Relative Clec7a expression. Data were log-transformed and analysed by Three way-ANOVA considering genotypes, sex and Plcg2 variants. As no sex effects were detected, data were consolidated into a Two way-ANOVA with post hoc multiple comparisons test using a two-stage step-up method controlling for FDR.* ***b*** *Relative IL6 (left) and Tnf (right) expression. Data were analysed by linear model and similarly to above, where a Plcg2 effect was seen and with no sex difference, the simple effect of Plcg2 variant on cytokine production was assessed (Bonferroni corrected).* ***c*** *Cytokine responses of hiPSC-derived microglial monocultures were assessed as described above. Each experiment consisted of 3 independent CRISPR-engineered clones for each genotype. Data in each experiment was normalised to the P522 response and the mean response for each clone was*

derived from the independent experiments. Data shown represent mean±SEM of the 3 independent clones of that genotype (n=3). Data were analysed by two-way ANOVA and where a significant effect of genotype was indicated, post-hoc Holm-Sidak tests were used to determine individual significant genotype effects. Significance between the 3 genotypes is indicated according to the figure key.

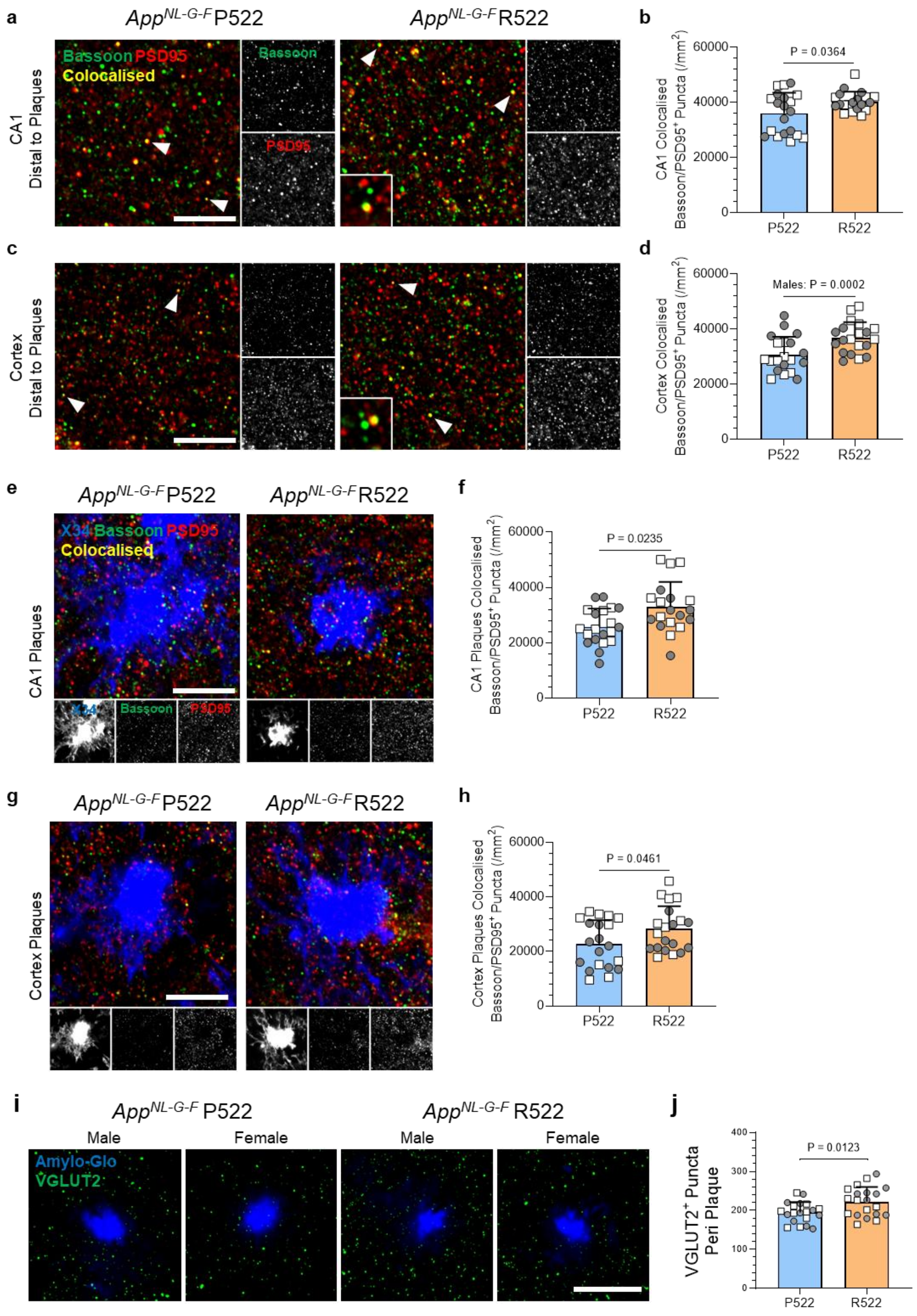

Supp Fig. 4. Plcg2^{R522} expression protects hippocampal and cortical synapses in App^{NL-G-F} mice. a *Representative hippocampal CA1 images showing presynaptic bassoon (green) and postsynaptic*

PSD95 (red) puncta located >30 µm from plaque material in mice carrying the risk P522 or protective R522 Plcg2 variants. Insets and arrows highlight colocalised puncta (yellow). Scale bar, 10 µm. ***b*** *Quantification of colocalised bassoon–PSD95 puncta in hippocampal CA1 >30 µm from plaques.* ***c*** *Representative cortical images showing bassoon (green) and PSD95 (red) puncta away from plaques. Insets and arrows highlight colocalised puncta (yellow). Scale bar, 10 µm.* ***d*** *Quantification of colocalised bassoon–PSD95 puncta in cortex >30 µm from plaques.* ***e*** *Representative hippocampal CA1 images of bassoon (green) and PSD95 (red) puncta within X34-positive peri-plaque regions (up to 5 µm from plaque border). Scale bar, 10 µm.* ***f*** *Quantification of colocalised bassoon–PSD95 puncta at hippocampal CA1 plaques.* ***g*** *Representative cortical images of bassoon (green) and PSD95 (red) puncta within peri-plaque regions (up to 5 µm from plaque border). Scale bar, 10 µm.* ***h*** *Quantification of colocalised bassoon–PSD95 puncta at cortical plaques.* ***i*** *Representative peri-plaque regions of interest (within 30 µm radius around plaque core) from $App^{NL\text{-}G\text{-}F}$ mice with the risk P522 and protective R522 Plcg2 variants stained for Amylo-Glo (blue) and VGLUT2 synaptic puncta (green). Scale bar 5 µm.* ***j*** *Quantification of VGLUT2 synaptic puncta peri-plaque. All data points represent individual mice (N = 10 males [squares] and 10 females [circles] per genotype; except panel f, where R522 females N = 9). Panels* ***b*** *and* ***d*** *represent mean puncta density in plaque-free tissue regions (>30 µm from plaques) within the CA1 stratum radiatum or cortical mid-layers. Panels* ***f*** *and* ***h*** *represent mean puncta density at matched plaque territories (up to 6 plaques per mouse). Data are summarised as mean ± SD. Statistical analyses (b, d, f, h) were performed using linear mixed-effects models including Plcg2 P522R variant and sex as fixed factors accounting for replicates per mouse. Reported P values correspond to the genotype fixed effect, except in panel d where a genotype-sex interaction was detected (male-specific genotype effect denoted). b Interaction P = 0.9753, Sex P = 0.6507, Genotype P = 0.0364. d Interaction P = 0.0130, Sex P = 0.9118, Genotype P = 0.0016. f Interaction P = 0.2000, Sex P = 0.2431, Genotype P = 0.0253. h Interaction P = 0.3852, Sex P = 0.1015, Genotype P = 0.0461. Data in j represented as the average of 5 plaque cores of similar sizes from the CA1 stratum radiatum. Data were analysed by Two-way ANOVA considering genotype and sex; no sex differences were detected for the datasets. P value reported represents the Two-way ANOVA effect of genotype.* ***b*** *Interaction P = 0.7130, Sex P = 0.8751, Genotype P =* 0.0123.

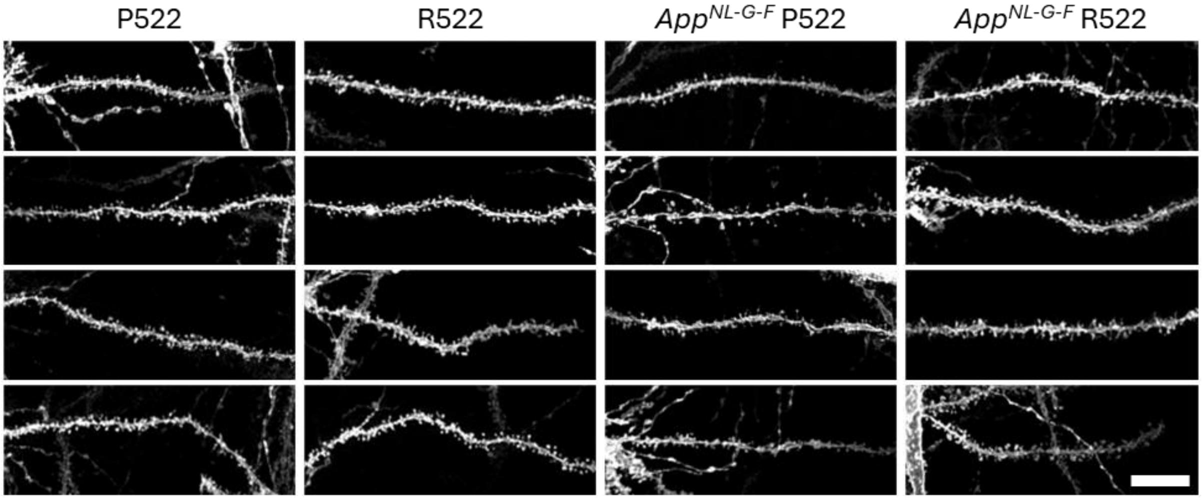

Supp Fig. 5. Examples of DiOlistic labelling of hippocampal dendritic spines. *Further representative DiOlistic labelled hippocampal CA1 dendrites with spine protrusions to complement the images shown in Fig. 5c. Scale bar 10 µm.*

Supp Video 1. Imaris rendered animation of peri-plaque engulfment of synaptic puncta in App$^{NL\text{-}G\text{-}F}$ mice. *Amylo-glo (blue), Iba1 (white), CD68 (green), PSD95 (red) and engulfed PSD95 (yellow). Scale bar 5 µm.*
